# Potential role of MRI to optimize clinical trial design for progressive supranuclear palsy and corticobasal degeneration

**DOI:** 10.1016/j.tjpad.2026.100486

**Published:** 2026-01-24

**Authors:** Jesús García-Castro, Lawren VandeVrede, Michael C. Donohue, Lídia Vaqué-Alcázar, Sara Rubio-Guerra, Judit Selma-González, Hilary W. Heuer, Alejandra O. Morcillo-Nieto, María Franquesa, Oriol Dols-Icardo, Alexandre Bejanin, Olivia Belbin, Juan Fortea, Daniel Alcolea, Maria Carmona-Iragui, Carla Abdelnour, Isabel Barroeta, Miguel Santos-Santos, María Belen Sánchez Saudinós, Isabel Sala, Alberto Lleó, Maria Luisa Gorno-Tempini, Maria Luisa Mandelli, Rema Raman, Anne-Marie A Wills, Eden Barragan, Irene Litvan, Brad Boeve, Brad Dickerson, Murray Grossman, Edward D. Huey, David J. Irwin, Alex Pantelyat, Carmela Tartaglia, Julio C. Rojas, Adam L. Boxer, Ignacio Illán-Gala, Four Repeat Tau Neuroimaging Initiative (4RTNI) and the AL108-231 Investigators

**Affiliations:** a Sant Pau Memory Unit, IR SANT PAU, Hospital de la Santa Creu i Sant Pau, Carrer de Sant Quintí, 77, 08041 Barcelona, Spain; b Centro de Investigación Biomédica en Red de Enfermedades Neurodegenerativas (CIBERNED), Avenida de Monforte de Lemos, 3-5, 28029, Madrid, Spain; c Departament de Medicina, Universitat Autònoma de Barcelona, 08193, Cerdanyola del Vallès, Spain; d Weill Institute for Neurosciences, Department of Neurology, Memory and Aging Center, University of California, 675 Nelson Rising Lane, Suite 190, San Francisco, CA 94143, USA; e Alzheimer Therapeutic Research Institute, Keck School of Medicine, University of Southern California, 9860 Mesa Rim Rd, San Diego, CA 92121, USA; f Department of Neurology, Massachusetts General Hospital, Harvard Medical School, 15 Parkman St, Boston, MA 02114, USA; g Parkinson and Other Movement Disorder Center, University of California, 9452 Medical Center Drive La Jolla, CA 92037, USA; h Department of Neurology, Mayo Clinic, Gonda Building, 200 1st St SW Floor 16, Rochester, MN 55905, USA; i Frontotemporal Disorders Unit, Departments of Neurology and Psychiatry, Massachusetts General Hospital and Harvard Medical School, 55 Fruit Street, Boston, MA 02114, United States; j Department of Neurology, Perelman School of Medicine at the University of Pennsylvania, 3400 Spruce St, Philadelphia, PA 19104, USA; k Department of Psychiatry and Human Behavior, Alpert Medical School of Brown University, 222 Richmond St, Providence, RI 02903, USA; l Department of Neurology, Johns Hopkins University School of Medicine, 733 N Broadway, Baltimore, MD 21205, USA; m Tanz Centre for Research in Neurodegenerative Diseases, University of Toronto, 60 Leonard Avenue, Toronto, Ontario, M5T 0S8, Canada

**Keywords:** Progressive supanuclear palsy, Corticobasal degeneration, Tauopathy, Magnetic resonance imaging, Biomarkers

## Abstract

**Background:**

Progressive supranuclear palsy (PSP) and corticobasal degeneration (CBD) are 4–repeat tauopathies (4RT) presenting with overlapping syndromes. Imperfect clinicopathological associations increase sample-size demands in clinical trials. We test whether MRI can enrich trials for PSP/CBD and provide sensitive MRI-based outcome measures.

**Methods:**

Longitudinal cohort analysis including participants from the 4 Repeat Tauopathy Neuroimaging Initiative (4RTNI) and phase 2/3 Davunetide trial (DAV). An MRI model trained on autopsy-confirmed cases predicted PSP (MRI-PSP) or CBD (MRI-CBD); corticobasal syndrome (CBS) with positive Alzheimer’s biomarkers was reclassified. Clinical scales and MRI-derived thickness/volume were analyzed with linear mixed-effects models. We derived data-driven MRI-signatures (optimal regional combinations) to minimize required trial sample sizes.

**Results:**

206 participants from 4RTNI (*n* = 106 with Richardson’s syndrome [RS], CBS, or nonfluent/agrammatic primary progressive aphasia [nfvPPA]) and DAV (*n* = 100 with RS). In 4RTNI, 49 participants were predicted MRI-PSP and 43 MRI-CBD. 76% of MRI-PSP had RS, 24% had CBS/nfvPPA; 66% of MRI-CBD had CBS. PSP and CBD signatures shared midbrain/pontine atrophy but differed in cortical involvement. PSP signature correlated strongly with 12-month change on the PSP Rating Scale (β = –0.59, *p* < 0.001). MRI-based signatures reduced the estimated sample sizes required to detect 30% reduction in progression over 12-months by 50% for MRI-PSP and 87% for MRI-CBD, compared with clinical outcomes. In DAV, feasibility was replicated.

**Conclusion:**

MRI-derived models can identify PSP or CBD with high accuracy, and MRI-based signatures track progression more sensitively than established clinical outcomes. Incorporating these tools into therapeutic trial design could reduce sample sizes and enable more inclusive disease-modifying trials for 4RT.

## Highlights

• MRI-based enrichment broadens trial selection for PSP and CBD.
• Data-driven MRI signatures provide sensitive and objective outcome measures.
• This method cuts PSP sample size by 50 % and CBD by 87 % vs clinical selection/endpoints.

## 1 Introduction

Progressive supranuclear palsy (PSP) and corticobasal degeneration (CBD) are primary 4-repeat tauopathies (4RT), defined by pathological aggregates of tau protein composed predominantly of proteoforms with four microtubule-binding domain repeats [1,2]. Although both conditions share the accumulation of 4RT as their underlying molecular pathology, they differ in their clinical presentations, anatomical distribution, and the ultrastructural features of the tau aggregates [2,3].

Traditionally, the clinical presentation of PSP was described as a rapidly progressive syndrome with early postural instability and vertical gaze palsy, now known as Richardson’s syndrome (RS) [4]. CBD was clinically described as an asymmetrical motor syndrome with cortical signs such as apraxia, cortical sensory loss, and the alien limb phenomenon, collectively referred to as corticobasal syndrome (CBS) [5]. However, subsequent clinico-pathological studies have demonstrated that 4RT pathology can give rise to a broad spectrum of motor, cognitive, and behavioral presentations that extend beyond these classical phenotypes [[6], [7], [8]].

Clinicopathological correlations in PSP and CBD are particularly limited in the early stages of the disease. Clinical syndromes associated with 4RT are often indistinguishable from those caused by other neurodegenerative pathologies, such as Alzheimer’s disease (AD) [9,10]. As a result, clinical trials have frequently equated RS with underlying PSP pathology, while CBD has been underrepresented or misclassified. This misalignment introduces clinical and biological heterogeneity in trial cohorts and may contribute to the failure of therapeutic efforts [11,12].

Robust biomarkers are urgently needed to enhance diagnostic accuracy and guide clinical trial design in 4RT. In this context, magnetic resonance imaging (MRI)-based models have emerged as promising tools [[13], [14], [15]]. Recent work has demonstrated that MRI features can accurately distinguish between PSP and CBD pathology in an autopsy-confirmed cohort across a wide range of clinical presentations [16]. These models could serve dual purposes: as enrichment strategies to select individuals with underlying 4RT pathology, and as sensitive markers for monitoring disease progression [6,13,14,17,18] and therapeutic response.

In this study, we aimed to evaluate the potential of MRI to improve clinical trial design in PSP and CBD. We hypothesized that MRI-derived diagnostic models would enable the selection of participants across a broader clinical spectrum with a greater likelihood of harboring underlying 4RT pathology, and that MRI-based measures would provide robust, quantitative outcomes to track disease progression over time.

## 2 Methods

### 2.1 Participants

**4RTNI sample**. The discovery cohort comprised participants recruited from seven sites—Massachusetts General Hospital (MGH), Johns Hopkins University, University of California San Diego (UCSD), University of Toronto, University of California San Francisco (UCSF), University of Pennsylvania, and Mayo Clinic in Rochester—as part of the 4 Repeat Tauopathy Neuroimaging Initiative (4RTNI). Individuals with RS fulfilled the National Institute of Neurological Disorders and Stroke and the Society for PSP, Inc. (NINDS-SPSP) criteria for RS [19]. In contrast, participants with CBS met criteria for possible or probable corticobasal syndrome associated with CBD pathology [7]. Non-fluent variant primary progressive aphasia (nfvPPA) was diagnosed according to consensus criteria [20]. Pathological data were available for 48 of the 106 4RTNI participants included (45 %).

**Davunetide trial sample (DAV)**. We also leveraged clinical and neuroimaging data from participants in the AL-108–231 phase 2/3 trial. This multicenter, double-blind, placebo-controlled study included participants with RS and did not demonstrate clinical efficacy of Davunetide over placebo after 52 weeks of follow-up [21].

For both samples, only participants with at least two clinical evaluations and two 3T-MRI scans passing quality control, with a minimum time delay of 4 months, were included. A flowchart of participant selection is presented in **Supplementary Materials:** Fig. 1.

**Fig. 1:**
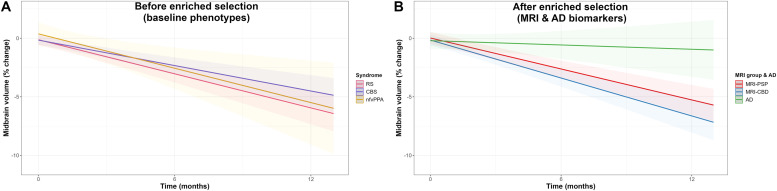
Volumetric change in the midbrain according to the selection strategy. Footnotes: Linear mixed-effects models showing the longitudinal percent change in midbrain volume according to clinical diagnosis only (A) and after the enriched selection using AD biomarkers and MLRM (B). Abbreviations: AD, Alzheimer’s disease; CBD, corticobasal degeneration; CBS, corticobasal syndrome; MRI, magnetic resonance imaging; nfvPPA, non-fluent variant of primary progressive aphasia; PSP, progressive supranuclear palsy; RS, Richardson’s syndrome.

**Clinical and neuroimaging outcomes.** The Progressive Supranuclear Palsy Rating Scale (PSPRS) [22] and the Schwab and England Activities of Daily Living scale (SEADL) were selected as clinical outcomes due to their availability in both cohorts and their frequent use as primary outcomes in previous trials. In 4RTNI, the disease severity was also rated using the modified PSPRS (mPSPRS) [23], Clinical Dementia Rating plus National Alzheimer’s Coordinating Center Frontotemporal lobar degeneration global score (CDR+NACC-FTLD-G), and sum of boxes (CDR+NACC-FTLD-SB) [24]. We also assessed percent change in midbrain volume, a widely used marker of disease progression in PSP [14,17,25].

**Evaluation of Alzheimer’s Disease.** In the 4RTNI cohort, a subset of participants underwent biomarker testing for AD, including amyloid-positron emission tomography (PET), tau-PET, and plasma pTau-217 (cut-off for positivity ≥0.25 pg/mL) [26]. Participants with CBS who tested positive for AD biomarkers were reclassified as having AD. When biomarker results were discordant, amyloid-PET status was used as the reference. According to previous reports, positive AD biomarkers in participants classified as RS were interpreted as a likely co-pathology [27,28].

**MRI Acquisition and Processing.** MRI for 4RTNI was obtained on 3T scanners—Siemens Tim Trio (UCSF, MGH), GE MR750 (UCSD) and GE Signa HDx (Toronto)—using 1 mm isotropic T1-weighted MPRAGE or IR-SPGR sequences (typical repetition time [TR]/echo time [TE]/inversion time [TI] ≈ 2.3 s/3 ms/900 ms or 7 ms/3 ms/400 ms). DAV trial scans were acquired on 48 scanners (1.5–3T) with a Mayo Clinic-standardised T1 protocol (MPRAGE or coronal/sagittal IR-SPGR). For homogenization purposes, only 3T scans from UCSF were included in the analyses of the DAV sample. Further acquisition details are provided in the **Supplementary Materials: Methods**. Cortical thickness, subcortical volumes, and total intracranial volume were estimated using *FreeSurfer* version 7.1.1 [29]. Brainstem segmentation was performed using a validated probabilistic atlas of the brainstem and its surrounding structures [30]. Mean cortical thickness was extracted for each region defined by the Desikan-Killiany atlas [31], and values were averaged across hemispheres. We performed additional analyses using ComBat harmonization [32] to ensure that variability related to scanners and acquisition protocols across sites did not bias our findings.

**Enrichment Strategy.** The likely underlying neurodegenerative etiology for each participant was determined using a previously validated model combining cortical and subcortical MRI-derived regions typically affected and spared, age, and biological sex [16]. This method has shown an accuracy of 92 % and 83 % in predicting underlying PSP and CBD, respectively, outperforming other classifiers. The optimal cut-off for predicted probability of PSP, distinguishing from CBD, was derived in the autopsy-confirmed subsample using the *cutpointr* R package [33] with 10,000 bootstrap iterations.

**Cross-sectional Analyses.** Baseline differences in demographic and clinical variables were assessed using the Kruskal–Wallis rank-sum test, Wilcoxon rank sum test or Fisher’s exact test, as appropriate. Statistical significance was set at *p* < 0.05, with the Bonferroni correction applied for multiple comparisons. Effect sizes were estimated using rank-biserial correlations.

**Longitudinal Analyses.** In the 4RTNI cohort, where follow-up intervals varied across participants, changes in clinical and imaging outcomes were analyzed using linear mixed-effects (LME) models. Time (in months) and diagnostic group (before vs. after the enrichment strategy) were modeled as fixed effects, with baseline age, biological sex, and the baseline value of each outcome included as covariates. Although modeled change is zero at baseline, including baseline outcome values helps control for between-subject variability at study entry and improves the estimation of longitudinal change. Interaction terms between group and time were tested, and random slopes were allowed. In the DAV cohort, where every participant had clinical and imaging assessments at baseline and at 52 weeks without missing data, changes in outcomes were analyzed using linear regression models. Change scores served as dependent variables, with diagnostic group, biological sex, baseline age, and baseline outcome values included as independent variables; baseline adjustment was again applied to control for between-subject variability. Interaction terms for diagnostic group were tested. Post-hoc pairwise comparisons of estimated marginal means (for the LME models) were adjusted using Bonferroni correction. Analyses were conducted in R (version 4.3.2) with the *lmerTest* [34], *ggeffects* [35], and *emmeans* packages.

**Sample Size Estimation for Hypothetical Trials.** Sample size estimates were calculated using the *longpower* package in R [36]. We estimated the number of participants required to detect a 30 % reduction in the rate of progression over 12 months, 80 % power and 0.05 alpha level, assuming a 10 % attrition rate. Models included time, biological sex, and baseline covariates as described above. Confidence intervals were computed using jackknife bootstrapping. Reported results refer to the total sample size (both arms).

**Data-driven Selection of MRI Outcomes.** We conducted a data-driven analysis to identify the optimal combination of cortical and subcortical regions most sensitive to change over a 12-month period. Multiple regional combinations showing longitudinal atrophy were tested using LME models and the *longpower* package [36]. To maintain computational feasibility and avoid overly complex or overfitted models, we restricted the search to every possible combination of up to five regions. This constraint also improves interpretability and ensures that selected outcomes remain practical for use in clinical trial settings. For each diagnostic group, we then identified the regional combination that yielded the smallest estimated sample size for detecting a hypothetical 30 % treatment effect over 12 months, with 80 % power, a two-sided α of 0.05, and a 10 % attrition rate.

**Clinical Relevance of MRI-derived Outcomes.** We evaluated the clinical relevance of the selected MRI-derived outcomes by computing the correlation with PSPRS, a score that has demonstrated clinical relevance in this population [37]. We fitted linear models adjusting for baseline age, PSPRS, and the corresponding cortical thickness and subcortical volume measures.

**Standard Protocol Approvals, Registrations, and Patient Consents.** The 4RTNI study was approved by the institutional review boards of UCSF, UCSD, Toronto, and MGH, with written informed consent obtained from all participants prior to enrollment. For the DAV cohort, ethical approval was granted by the local ethics committees at each participating site, and all participants provided written informed consent at the time of recruitment in accordance with local regulations.

**Data availability.** The datasets analyzed in this study are available upon reasonable request.

## 3 Results

**Optimal cut-off point determination for the enrichment strategy.** In the subgroup of 4RTNI participants with autopsy-confirmed diagnoses (*n* = 48), we determined the optimal cut-off for the predicted probability of underlying PSP pathology using a previously defined multinomial logistic regression model that discriminates PSP from CBD [16]. The optimal cut-off was 0.11 (AUC 0.90, sensitivity 0.80, specificity 0.92, positive predictive value 0.97, negative predictive value 0.63) (**Supplementary Materials:** Fig. 2). We also tested a model that discriminated between PSP and any other pathology, showing an optimal cut-off of 0.17 (AUC 0.88, sensitivity 0.74, specificity 0.92, positive predictive value 0.96, negative predictive value 0.57). The first model was selected because it provided a better performance and most closely matched the characteristics of our study cohort. Details of the pathologically confirmed diagnoses are shown in **Supplementary Materials:** Table 1.

**Fig. 2:**
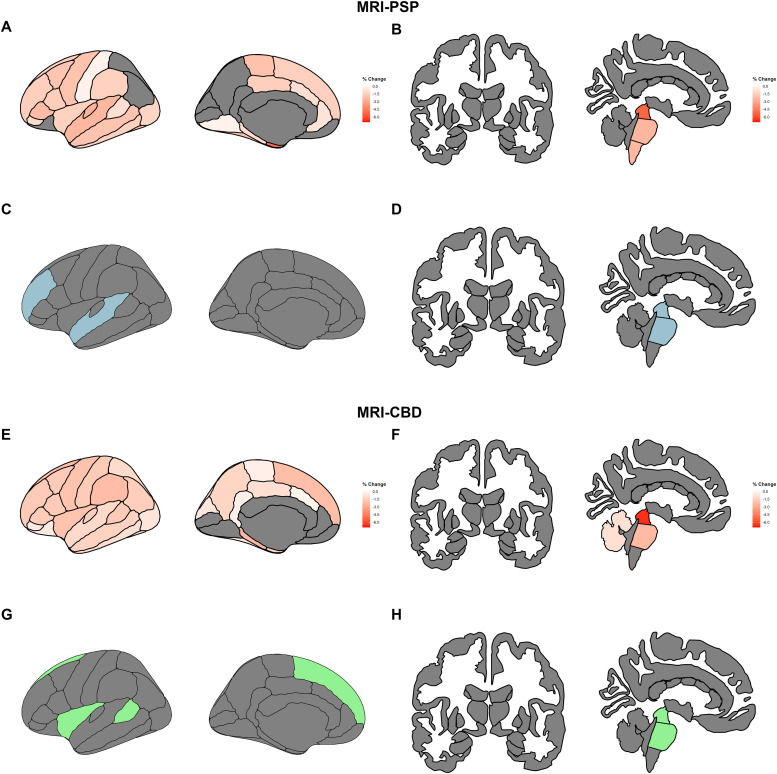
Imaging changes at 12 months and data-driven MRI-signatures. Footnotes: Predicted changes in cortical thickness and subcortical volumes at 12 months in MRI-PSP (A-B) and MRI-CBD (E-F). Values were derived from linear mixed-effects models. The most efficient data-driven combination of regions of interest to detect 30 % reduction in atrophy with 10 % attrition rate is shown for MRI-PSP (C-D) and MRI-CBD (G-H). Abbreviations: CBD, corticobasal degeneration; MRI, magnetic resonance imaging; PSP, progressive supranuclear palsy.

**Table 1:** Sample characteristics.

	4RTNI					DAV			
Characteristic	All, *N* = 106	MRI-PSP, *N* = 51	MRI-CBD, *N* = 41	AD, *N* = 14	Adjusted p-value	All, *N* = 100	MRI-PSP, *N* = 85	MRI-CBD, *N* = 15	p-value
**Age**	68 (8)	70 (8)	65 (9)	67 (10)	>0.9	67 (7)	68 (6)	61 (8)	**0.003**
**Biological Sex**					>0.9				0.2
Woman	53 (50 %)	31 (61 %)	17 (41 %)	5 (36 %)		54 (54 %)	48 (56 %)	6 (40 %)	
Man	53 (50 %)	20 (39 %)	24 (59 %)	9 (64 %)		46 (46 %)	37 (44 %)	9 (60 %)	
**Number of visits**					>0.9				
2	23 (22 %)	16 (31 %)	5 (12 %)	2 (14 %)		100 (100 %)	85 (100 %)	15 (100 %)	
3	63 (59 %)	25 (49 %)	29 (71 %)	9 (64 %)					
>3	20 (19 %)	10 (20 %)	7 (17 %)	3 (21 %)					
**Clinical phenotype**					**<0.001**				
RS	47 (44 %)	39 (76 %)	8 (20 %)	0 (0 %)		100 (100 %)	85 (100 %)	15 (100 %)	
CBS	51 (48 %)	12 (24 %)	27 (66 %)	12 (86 %)					
nfvPPA	8 (8 %)	0 (0 %)	6 (14 %)	2 (14 %)					
**Years of symptoms**					>0.9				>0.9
≤ 5 Years	75 (72 %)	39 (72 %)	36 (72 %)	75 (72 %)		86 (95 %)	72 (94 %)	14 (100 %)	
> 5 Years	29 (28 %)	15 (28 %)	14 (28 %)	29 (28 %)		5 (5 %)	5 (6 %)	0 (0 %)	
**PSPRS**	26 (15)	33 (14)	21 (13)	15 (11)	**<0.001**	38 (12)	40 (11)	29 (18)	**0.003**
**SEADL**	63 (24)	59 (25)	66 (22)	70 (21)	>0.9	54 (25)	51 (24)	71 (26)	**0.005**
**Midbrain volume**	5404 (800)	4826 (536)	5940 (618)	5936 (604)	**<0.001**	4836 (683)	4695 (553)	5630 (819)	**<0.001**
**Stability of MRI-based classification**					**<0.001**				**<0.001**
CBD to PSP	14 (13 %)	0 (0 %)	14 (34 %)	0 (0 %)		4 (4 %)	0 (0 %)	4 (27 %)	
PSP to CBD						1 (1 %)	1 (1 %)	0 (0 %)	
Stable	92 (87 %)	51 (100 %)	27 (66 %)	14 (100 %)		95 (95 %)	84 (99 %)	11 (73 %)	

**Footnote:** values reported are mean (standard deviation) or n ( %). Bonferroni correction for multiple testing was applied in 4RTNI.

**Abbreviations:** AD, Alzheimer’s disease; CBD, corticobasal degeneration; CBS, corticobasal syndrome; MRI, magnetic resonance imaging; nfvPPA, non-fluent variant of primary progressive aphasia; PSP, progressive supranuclear palsy; PSPRS, progressive supranuclear palsy rating scale; RS, Richardson’s syndrome; SEADL, Schwab and England Activities of Daily Living scale.

**Baseline demographic and clinical data.** The 4RTNI cohort included 106 individuals (50 % female), with a mean (SD) age of 68 (8) years. Among them, 47 (44 %) had a diagnosis of RS, 51 (48 %) of CBS, and 8 (7.5 %) of nfvPPA. Of the 51 CBS participants, 12 (29 %) had AD pathology based on AD biomarkers. Table 1 and **Supplementary Materials: Table 2** present the baseline characteristics and participant distribution after the AD biomarker and MRI-based enrichment strategy, with 51 individuals predicted to have PSP (MRI-PSP) and 41 predicted to have CBD (MRI-CBD). Notably, of the 51 participants predicted to have PSP pathology, 39 (76 %) were clinically diagnosed with RS, and the remaining 12 (24 %) with CBS. Among the 41 participants predicted to have CBD pathology, 27 (66 %) were diagnosed with CBS and 8 (20 %) with RS. In the pre-enrichment groups, 31 (67 %) RS participants had symptoms for less than 5 years, and 16 (35 %) for less than 3 years, while 38 CBS participants (76 %) had symptoms for under 5 years, and 23 (46 %) for under 3 years. Symptom duration was comparable in the biomarker-enriched cohort. The MRI-PSP group showed similar proportions, with 37 (73 %) participants symptomatic for under 5 years and 18 (35 %) for under 3 years. Likewise, the MRI-CBD group included 26 of 39 participants (67 %) with symptoms for less than 5 years and 18 (46 %) for less than 3 years.

At baseline, MRI-PSP participants had higher PSPRS than those with MRI-CBD (*r* = 0.48, 95 % CI: 0.63, 0.59, *p* < 0.001) and AD (*r* = 0.76, 95 % CI: 0.64, 0.84, *p* < 0.001). Mean (SD) PSPRS were 33 [18] for MRI-PSP, 21 [13] for MRI-CBD, and 15 [11] for AD. MRI-PSP participants also showed higher mPSPRS compared to MRI-CBD (*r* = 0.61, 95 % CI: 0.49, 0.70, *p* < 0.001) and AD (*r* = 0.83, 95 % CI: 0.74, 0.89, *p* < 0.001). Mean (SD) mPSPRS were 7.3 (4.5), 3.0 (3.7), and 1.5 (1.5), respectively. There were no other significant group differences in SEADL, MoCA, CDR+NACC-FTLD-G, or CDR+NACC-FTLD-SB. Mean (SD) midbrain volume at baseline was 4826 (536) cc in MRI-PSP, 5940 (618) cc in MRI-CBD, and 5936 (604) cc in AD. Pairwise comparisons showed that midbrain volume was smaller in MRI-PSP compared to both MRI-CBD (*r* = −0.82, 95 % CI: −0.87, −0.77, *p* < 0.001) and AD (*r* = −0.84, 95 % CI: −0.90, −0.76, *p* < 0.001). Notably, all participants categorized as MRI-PSP were consistently classified as such in the follow-up scans.

Baseline characteristics according to clinical diagnosis are shown in **Supplementary Materials: Table 3**. As expected, participants with RS presented with higher PSPRS at baseline compared to both CBS (*r* = 0.47, 95 % CI: 0.35, 0.59, *p* < 0.001) and nfvPPA (*r* = 0.93, 95 % CI: 0.87, 0.96, *p* < 0.001). Mean (SD) values for PSPRS of 33 [15] in RS, 23 [12] in CBS, and 8 [5] in nfvPPA. RS participants also showed higher mPSPRS scores at baseline compared to both CBS (*r* = 0.60, 95 % CI: 0.49, 0.69, *p* < 0.001) and nfvPPA (*r* = 0.90, 95 % CI: 0.82, 0.94, *p* < 0.001). Mean (SD) mPSPRS were 7.5 (4.7), 3.1 (3.3), and 0.6 (0.7), respectively.

The DAV cohort included 100 participants (56 % female) with a diagnosis of RS and a mean (SD) age at baseline of 67 (7) years. After the MRI-based enrichment strategy, 85 individuals (85 %) were deemed to have PSP (MRI-PSP), and 15 (15 %) to have CBD (MRI-CBD) at baseline. Mean (SD) PSPRS at baseline was 40 (11) in MRI-PSP and 29 (18) in MRI-CBD (*r* = 0.40, 95 % CI: 0.19, 0.57, *p* = 0.003). Mean (SD) SEADL was 51 (24) in MRI-PSP and 71 (26) in MRI-CBD (*r* = −0.37, 95 % CI: −0.55, −0.16, *p* = 0.005). As expected, participants with MRI-PSP had lower midbrain volumes at baseline compared to MRI-CBD: 4695 (553) cc vs 5630 (819) cc (*r* = −0.64, 95 % CI: −0.76, −0.49, *p* < 0.001). Following biomarker-enriched selection, symptom duration was similar across groups, with 94 % of participants in the MRI-PSP group (*n* = 72) and 100 % in the MRI-CBD group (*n* = 14) presenting with symptoms for less than 5 years (*p* < 0.001; Table 1).

**Longitudinal clinical and neuroimaging data.** In the 4RTNI cohort, clinically defined groups showed the following 12-month PSPRS increases estimated by LME models: RS, 13.04 points (95 % CI, 9.03–17.05); CBS, 7.30 (95 % CI, 3.52–11.09); and nfvPPA, 10.71 (95 % CI, 1.22–20.21). The rate of PSPRS progression was greater in RS compared with CBS (β = −0.473; 95 % CI, −0.941 to −0.005; *p* = 0.050). The estimated 12-month percent reduction in midbrain volume was −5.95 % (95 % CI, −7.34 to −4.55) in RS, −4.51 % (95 % CI, −5.86 to −3.15) in CBS, and −5.50 % (95 % CI, −9.08 to −1.91) in nfvPPA, with no significant between-group differences in atrophy rate (Fig. 1**A**).

Considering biomarker-enriched groups, the estimated 12-month PSPRS increases were 11.91 points (95 % CI, 7.99–15.83) for MRI-PSP, 8.37 (95 % CI, 4.13–12.61) for MRI-CBD, and 8.54 (95 % CI, 1.22–15.86) for AD, with no significant differences in progression rates. The corresponding percent reductions in midbrain volume were −5.27 % (95 % CI, −6.55 to −3.99) for MRI-PSP, −6.63 % (95 % CI, −8.06 to −5.20) for MRI-CBD, and −0.94 % (95 % CI, −3.30 to 1.41) for AD. The AD group had a significantly lower atrophy rate (β = 0.379; 95 % CI, 0.143–0.615; *p* = 0.002) and higher 12-month midbrain volumes than MRI-PSP (*p* = 0.006) and MRI-CBD (*p* < 0.001) (Fig. 1**B**).

**Fig. 3:**
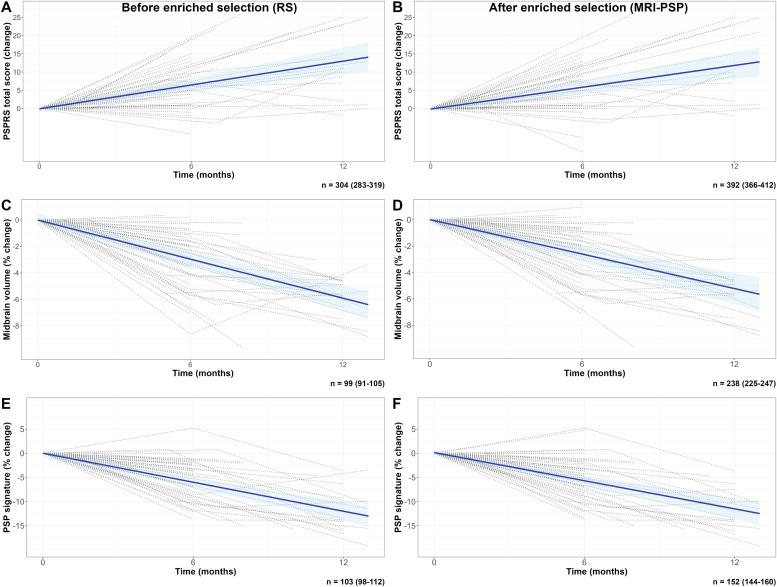
Longitudinal clinical and MRI change for RS and MRI-PSP groups. Footnotes: Panels show the predicted change in PSPRS (A,B), midbrain volume reduction (C,D), and the PSP signature (E,F), derived from linear mixed-effects models before enriched selection (inclusion of all participants with RS) and after enriched selection (inclusion of all participants with predicted PSP by MRI [MRI-PSP], regardless of their clinical phenotype). Individual trajectories for each variable are superimposed (dotted lines). The bootstrapped estimated sample size for a clinical trial (30 % reduction at 12 months with 10 % attrition) is displayed at the bottom of each panel. PSPRS was selected among the clinical outcomes because it showed the best sample size estimates in this population. Abbreviations: MRI, magnetic resonance imaging; PSP, progressive supranuclear palsy; PSPRS, Progressive supranuclear palsy rating scale; RS, Richardson’s syndrome.

Across the other clinical outcomes, progression rates did not differ among diagnostic categories in both the clinically defined and biomarker-enriched groups. However, in the RS subgroup, mPSPRS increased faster compared to CBS (β = −0.226, 95 %CI: −0.390– −0.061, *p* = 0.009), whereas in participants with predicted AD, it was slower than the other biomarker-enriched subgroups (β = −0.282, 95 %CI: −0.529– −0.035, *p* = 0.027). Detailed information about longitudinal outcomes is shown in **Supplementary Materials: Tables 4–6** and Fig. 3.

In DAV, no significant differences were observed in the rate of progression, as measured by PSPRS, between the MRI-PSP and MRI-CBD groups (β = 0.084, 95 %CI: −0.376–0.544, *p* = 0.721). The midbrain atrophy rate did not differ between groups either (β = 0.076, 95 %CI: −0.062–0.214, *p* = 0.285) (**Supplementary Materials: Table 7**).

In 4RTNI, MRI-PSP and MRI-CBD showed partially overlapping cortical and brain-stem changes at month 12 (Fig. 2**, Supplementary Materials: Tables 8–9**). However, differences in longitudinal imaging changes were noted depending on the classification scheme (clinically defined or biomarker-enriched groups, **Supplementary Materials:** Figs. 4**–5, Tables 10–11)**. A similar cortical and brainstem pattern of changes was observed in the DAV dataset (**Supplementary Materials: Fig. 5**).

**Fig. 4:**
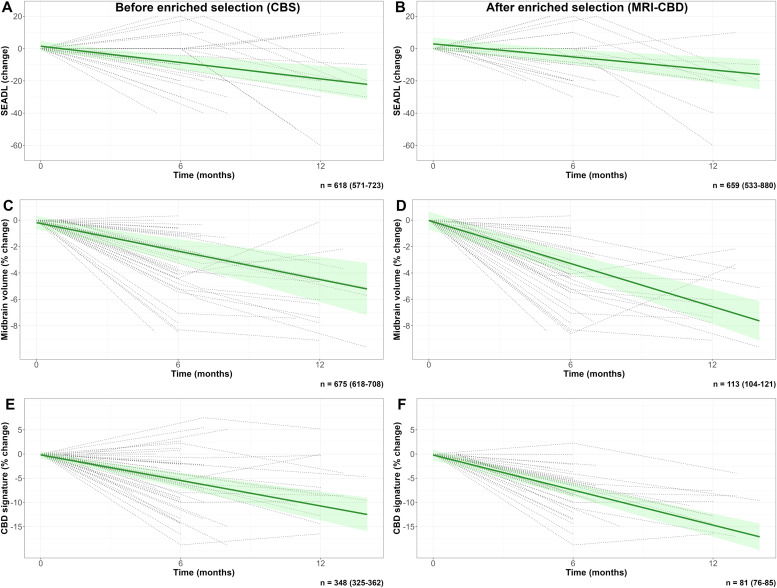
Longitudinal clinical and MRI change for CBS and MRI-CBD groups. Footnotes: Panels show the predicted clinical change as measured by SEADL (A,B), midbrain volume reduction (C,D), and percent change for the CBD signature (E,F), derived from linear mixed-effects models before enriched selection (inclusion of all participants with CBS) and after enriched selection (inclusion of all participants with predicted CBD by MRI [MRI-CBD], regardless of their clinical phenotype). Individual trajectories for each variable are superimposed (dotted lines). The bootstrapped estimated sample size for a clinical trial (30 % reduction at 12 months with 10 % attrition) is displayed at the bottom of each panel. SEADL was selected among the clinical outcomes because it showed the best sample size estimates in this population. Abbreviations: MRI, magnetic resonance imaging; CBD, corticobasal degeneration; CBS, corticobasal syndrome; SEADL, Schwab and England Activities of Daily Living scale.

**Data-driven selection of MRI signature.** We applied a data-driven approach in the 4RTNI sample to identify the optimal MRI-based signature for tracking brain changes at month 12 (Fig. 2). For participants with predicted PSP (MRI-PSP), the combination of changes in midbrain and pons volumes, as well as superior temporal and rostral middle frontal thickness, was the most powerful signature. For participants with predicted CBD (MRI-CBD), the combination of changes in the midbrain, pons, superior frontal, insula, and banks of the superior temporal sulcus was the most relevant signature. Notably, the change in the data-driven MRI-derived PSP signature showed a statistically significant correlation with the change in PSPRS (*p* < 0.001, β = −0.590, adjusted R² = 0.341). A significant correlation was also found between the CBD signature and PSPRS change (*p* < 0.001, β = −0.515, adjusted R² = 0.364) (**Supplementary Materials: Fig. 7**). The models that included the data-driven MRI-based signatures explained more variance in the change in PSPRS compared to the models that tracked only midbrain volume, both in PSP (*p* < 0.001, β = −0.524, adjusted R² = 0.245) and CBD participants (*p* < 0.001, β = −0.508, adjusted R² = 0.332).

**Sample size estimation for hypothetical clinical trials.** For PSP, a trial restricted to participants with the RS phenotype and powered to detect a 30 % reduction in PSPRS at 12 months would require 304 participants for both arms (95 % CI: 283–319). Using imaging outcomes reduced sample size requirements to 99 (95 % CI: 91–105) for midbrain atrophy and 103 (95 % CI: 98–112) for the MRI-derived PSP signature (Fig. 3). When participants were selected based on MRI evidence of PSP (MRI-PSP), sample size estimates increased to 392 (95 % CI: 366–412) for PSPRS, but decreased substantially when imaging outcomes were used: 238 (95 % CI: 225–247) for midbrain atrophy and 152 (95 % CI: 144–160) for the MRI-derived outcome.

For CBD, a trial including CBS participants and using SEADL as the primary outcome would require 618 participants across both arms (95 % CI: 571–723). Imaging outcomes offered limited efficiency gains in this clinically defined cohort: 675 (95 % CI: 618–708) for midbrain atrophy and 348 (95 % CI: 325–362) for the MRI-derived CBD outcome (Fig. 4). In contrast, when enrichment was based on MRI and AD biomarkers to define MRI-CBD, sample size requirements dropped sharply: from 659 (95 % CI: 533–880) with SEADL to 113 (95 % CI: 104–121) with midbrain atrophy and only 81 (95 % CI: 78–85) with the MRI-derived outcome (Fig. 4).

The estimated sample sizes for the other available clinical scores, along with detailed information about the data-driven MRI signatures across groups, are shown in **Supplementary Materials: Figs. 8–9** and **Supplementary Materials: Tables 12–**15. The analyses performed after ComBat harmonization showed remarkably similar results (**Supplementary Materials: Tables 16–17**).

To ensure that the observed sample size reductions associated with the enriched selection strategy and the data-driven MRI signature were not driven by participants at advanced disease stages, we conducted a sensitivity analysis excluding individuals with more than 5 years of symptom duration (**Supplementary Materials: Table 18**). When restricting the analyses to this subsample, reduced estimated sample sizes were still observed if using the enriched selection strategy and the data-driven MRI signatures: 288 (95 % CI: 261–311) participants when including RS individuals and using PSPRS as the outcome, versus 189 (95 % CI: 177–197) MRI-PSP participants when using the MRI-derived outcome. For CBS, the estimated sample size was 535 (95 % CI: 482–660) when using SEADL, compared with 76 MRI-CBD participants (95 % CI: 68–83) when using the MRI-derived outcome.

We replicated the feasibility of using MRI signatures as outcome measures in hypothetical clinical trials in the DAV dataset under the same assumptions. Detecting the prespecified reduction in PSPRS among RS participants required an estimated sample size of 147 (95 % CI: 141–150), whereas using the MRI-based PSP signature as the outcome yielded an estimate of 171 (95 % CI: 164–175). Restricting inclusion to participants with a high MRI-based probability of PSP (MRI-PSP) resulted in comparable sample size estimates for PSPRS (150; 95 % CI: 143–154) and the MRI signature (146; 95 % CI: 140–150). In contrast, imaging measures exhibited greater variability in the DAV dataset, particularly when progression was assessed using a single region such as the midbrain, leading to substantially higher sample size requirements for both RS (343; 95 % CI: 317–354) and MRI-PSP (302, 95 % CI: 290–328). Inclusion of lower-quality 1.5T MRI data, despite ComBat harmonization, further increased overall sample size estimates (**Supplementary Materials: Table 19, Fig. 10**).

## 4 Discussion

In this multicenter study spanning two independent datasets, we demonstrated that a two-step, MRI-guided strategy—(i) baseline diagnostic enrichment and (ii) longitudinal MRI-based outcome measures—substantially improves the statistical power and feasibility of disease-modifying trials in PSP and CBD. Baseline enrichment identified participants with high etiological probability irrespective of clinical presentation, and data-driven longitudinal measures combining brainstem volumes with cortical thickness in different regions were more sensitive to change than traditional clinical scales.

We applied an MRI-based logistic-regression model previously trained on autopsy-confirmed cases [16] to classify participants as having a high probability of PSP versus CBD in a clinical setting where 4RT are common. This MRI-based enrichment captured individuals with a high probability of PSP or CBD presenting as RS, CBS of nfvPPA. We then derived data-driven MRI signatures that tracked disease progression more sensitively than established clinical scales or single-region volumetry. Coupling these two steps sharply reduced the theoretical sample size of hypothetical clinical trials by up to 50% for PSP and 87% for CBD.

A key finding was that 24 % of participants predicted to have PSP (MRI-PSP) did not fulfill the canonical RS criteria at enrolment. Conversely, 15 % of canonical RS cases in the Davunetide trial were predicted to harbour CBD pathology. These findings align with those reported in large autopsy series and highlight the limitations of assuming *a priori* that RS is equivalent to PSP [12,38]. Excluding non-RS phenotypes from therapeutic trials may preclude the inclusion of a significant proportion of patients with PSP at early stages of the disease, presenting as nfvPPA [39] or CBS [10]. Equally important, including misclassified CBD cases in a PSP-targeted trial may dilute efficacy signals if a therapy acts selectively on PSP molecular conformers [3]. Stratification by baseline MRI prediction, therefore, offers a safeguard against pathological admixture/biological heterogeneity.

Recalibration of cut-offs for the logistic regression model was necessary to maintain accuracy in our 4RT-enriched cohort, underscoring that decision thresholds should not be copied blindly between studies conducted in different clinical contexts. Furthermore, additional research is necessary to validate cut-offs in larger prodromal PSP populations. Previous studies have validated alternative imaging markers such as the Magnetic Resonance Parkinsonism Index [13,18] or the Automated Imaging Differentiation for Parkinsonism [15] to distinguish RS from Parkinson’s disease. However, these imaging markers have not been validated to predict PSP and CBD across other phenotypes that are less frequently encountered in movement disorders clinics [12] and have shown limited accuracy in distinguishing between PSP and CBD [16].

While midbrain atrophy remains a robust hallmark of 4RTs [17], our data-driven approach identified composite signatures that captured change more efficiently in both PSP and CBD. For MRI-PSP, a combination of midbrain and pontine volumes, together with superior temporal and rostral middle frontal cortical thickness, provided the strongest longitudinal signal. For MRI-CBD, the optimal signature included midbrain, pons, insula, banks of the superior temporal sulcus and superior frontal thickness. These signatures correlated with longitudinal PSPRS changes and cut the required sample size by half relative to PSPRS alone. The superior performance of multiregional composites likely reflects the heterogeneous spatial distribution of tau pathology [40], combined with reduced measurement noise in small or irregular structures [41,42]. Overall, our findings contribute to the existing evidence supporting the use of MRI-based methods to increase diagnostic confidence and track disease progression in PSP [14]. It is worth noting that a proportion of participants classified as having a high probability of CBD on the first MRI scan were reclassified as MRI-PSP on the subsequent scan. However, the persistence of the estimated sample size reduction in the sensitivity analyses excluding participants with more than five years of symptoms reinforces that the beneficial effect of the enriched selection strategy and the use of the MRI signature as outcome is not driven by late-stage atrophy. This supports that the sample size reductions observed would also be achievable in clinical trials enrolling patients at earlier disease stages.

Taken together, MRI-based enrichment and MRI progression signatures may allow the design of leaner, shorter, and more inclusive trials. In the 4RTNI cohort, selecting MRI-PSP participants and using the MRI-based PSP signature as an outcome would require only 152 participants for both arms to detect a 30% treatment effect over 12 months, compared to 304 with PSPRS alone. For CBD, coupling the MRI-CBD screen with the MRI-based CBD signature reduced the total sample size from 618 to 81 participants. This represents an eight-fold reduction, pushing CBD trials firmly into the realm of feasibility.

Scanner protocol heterogeneity remains a challenge, as illustrated by the noisier longitudinal measurements in the Davunetide dataset. FreeSurfer quantifications are generally reliable [41], but their use is time-consuming, computationally intensive, and requires domain expertise, which may limit scalability in large or multi-centre trials. While the latter concerns remain, processing time may be mitigated by the wider adoption of accelerated neuroimaging pipelines such as FastSurfer, which substantially reduce post-processing duration [43]. Measurement variance in small, irregular structures such as the entorhinal cortex and amigdala [42], blunts their longitudinal signal. Likewise, the striatum, prominently atrophic in voxel-based PSP/CBD studies [17] did not emerge as a top change-sensitive region, likely because atlas-based segmentation becomes erratic when neighbouring tissue is affected, creating spurious volume fluctuations [44]. Validation in independent clinical cohorts is warranted, as sample-size estimates obtained without cross-validation may be overly optimistic [45]. Standardisation around harmonised 3T protocols, central quality control, and modern denoising pipelines will be essential for prospective trials that intend to use MRI as both a screening tool and an outcome measure. Additional analyses are needed for modern trials (i.e, NCT06355531) to optimize MRI-derived signatures tracking disease progression in PSP and CBD. A further strategy to address intercenter variability will be the exploration of z-score–based normalization of cortical and subcortical measures using site-specific distributions to mitigate scanner- or protocol-related differences. As most clinical samples will lack pathological confirmation and optimal thresholds are likely to depend on clinical context and cohort composition [46], a logical next step would be to derive data-driven cutoffs using Gaussian mixture models, which can flexibly model latent disease subpopulations and provide probabilistic classifications that are more robust to inter-site and inter-individual variability. To this end, we have developed an open-access ShinyApp that enables users to derive site-specific cutoffs by inputting cortical thickness and subcortical volume measures (https://pspredictor.shinyapps.io/pspredictor/).

Although our findings are encouraging, several caveats warrant discussion. First, the majority of participants lacked neuropathological confirmation; however, a sizable autopsy subset allowed us to recalibrate the MRI-based logistic model against proven pathology and apply the refined cut-off in the independent Davunetide cohort, thereby preserving diagnostic accuracy across datasets. We acknowledge that atypical or mixed-pathology cases remain challenging to classify in the absence of definitive tissue confirmation, and that expanding the number of autopsy-proven cases would strengthen future validations. Second, we did not integrate emerging fluid biomarkers, such as plasma phosphorylated tau-217 or tau/TAR DNA-binding protein 43 species in small extracellular vesicles, which hold promise for identifying 4RT [47]. Yet, by relying exclusively on standard structural MRI, we provide an immediately deployable framework for multicentre trials, while leaving room for a future two-stage algorithm in which biofluid screening could precede MRI subtyping. Third, we interpreted reduced brain atrophy as a beneficial treatment effect; however, MRI-detected changes do not necessarily translate into clinically meaningful improvement [48]. Fourth, despite stringent probability thresholds, the MRI-CBD group may still contain a small proportion of non-4RT pathologies, such as Lewy body disease, reflecting a current limitation of MRI-only enrichment and underscoring the potential role of α-synuclein biomarkers in future screening [49]. Nonetheless, the uniform midbrain atrophy pattern in this cohort suggests that most individuals do harbor underlying 4RT. Finally, the study included relatively few participants with nfvPPA, which limited phenotype-specific inferences. That said, the successful inclusion and classification of these cases illustrate the potential generalizability of the enrichment strategy beyond the canonical motor presentations, paving the way for larger, dedicated studies currently underway. Extending this MRI-based enrichment classification approach to other 4RT phenotypes (PSP-parkinsonism, PSP-progressive gait freezing, PSP-ocular motor dysfunction, PSP-postural instability of frontal behavioral-spatial syndrome) will be an essential next step for capturing the full clinical variability that can be expected in these diseases. While our work focused on a cohort defined by the NINDS-SPSP criteria [19], the broader phenotype coverage of the 2017 Movement Disorders Society diagnostic criteria [6] would allow future studies to extend this methodology to more heterogeneous PSP populations.

## 5 Conclusion

MRI might play a dual role as both a scalable screening tool and a sensitive outcome measure in PSP and CBD. By embracing MRI-guided enrichment and composite progression signatures, forthcoming disease-modifying trials can be shorter and smaller while capturing a broader range of clinical presentations across the 4RT spectrum.

## Declaration of generative AI and AI-assisted technologies in the writing process

During the preparation of this work the author used ChatGPT solely to refine English wording and translation. After using this tool, the author reviewed and edited the content as needed and takes full responsibility for the content of the published article.

## CRediT authorship contribution statement

**Jesús García-Castro:** Writing – original draft, Software, Formal analysis, Conceptualization. **Lawren VandeVrede:** Writing – review & editing, Data curation. **Michael C. Donohue:** Writing – review & editing, Methodology, Conceptualization. **Lídia Vaqué-Alcázar:** Writing – review & editing. **Sara Rubio-Guerra:** Writing – review & editing. **Judit Selma-González:** Writing – review & editing. **Hilary W. Heuer:** Writing – review & editing. **Alejandra O. Morcillo-Nieto:** Writing – review & editing. **María Franquesa:** Writing – review & editing. **Oriol Dols-Icardo:** Writing – review & editing. **Alexandre Bejanin:** Writing – review & editing. **Olivia Belbin:** Writing – review & editing. **Juan Fortea:** Writing – review & editing. **Daniel Alcolea:** Writing – review & editing. **Maria Carmona-Iragui:** Writing – review & editing. **Carla Abdelnour:** Writing – review & editing. **Isabel Barroeta:** Writing – review & editing. **Miguel Santos-Santos:** Writing – review & editing. **María Belen Sánchez Saudinós:** Writing – review & editing. **Isabel Sala:** Writing – review & editing. **Alberto Lleó:** Writing – review & editing. **Maria Luisa Gorno-Tempini:** Writing – review & editing. **Maria Luisa Mandelli:** Writing – review & editing. **Rema Raman:** Writing – review & editing. **Anne-Marie A Wills:** Writing – review & editing, Data curation. **Eden Barragan:** Writing – review & editing. **Irene Litvan:** Writing – review & editing, Data curation. **Brad Boeve:** Writing – review & editing, Data curation. **Brad Dickerson:** Writing – review & editing, Data curation. **Murray Grossman:** Data curation. **Edward D. Huey:** Writing – review & editing, Data curation. **David J. Irwin:** Writing – review & editing, Data curation. **Alex Pantelyat:** Writing – review & editing, Data curation. **Carmela Tartaglia:** Writing – review & editing. **Julio C. Rojas:** Writing – review & editing, Data curation. **Adam L. Boxer:** Writing – review & editing, Data curation. **Ignacio Illán-Gala:** Writing – original draft, Supervision, Formal analysis, Conceptualization.

## Declaration of competing interest

The authors declare the following financial interests/personal relationships which may be considered as potential competing interests:

Ignacio Illan-Gala reports financial support was provided by Carlos III Health Institute. Jesus Garcia-Castro reports financial support was provided by Carlos III Health Institute. Ignacio Illan-Gala reports financial support was provided by Alzheimer’s Association. M.C. Donohue reports financial support was provided by National Institutes of Health. M. Carmona-Iragui reports financial support was provided by Global Brain Health Institute. M. Carmona-Iragui reports financial support was provided by Jérôme Lejeune Foundation. M. Carmona-Iragui reports financial support was provided by Alzheimer’s Association. L. Vaque-Alcazar reports financial support was provided by Carlos III Health Institute. S. Rubio-Guerra reports financial support was provided by Alzheimer’s Association. A. Bejanin reports financial support was provided by Carlos III Health Institute. A. Bejanin reports financial support was provided by Alzheimer’s Association. O. Dols-Icardo reports financial support was provided by Spanish Foundation for the Promotion of Amyotrophic Lateral Sclerosis Research. O. Dols-Icardo reports financial support was provided by Alzheimer’s Association. O. Dols-Icardo reports financial support was provided by Jérôme Lejeune Foundation. Daniel Alcolea reports financial support was provided by Carlos III Health Institute. I. Illan-Gala reports financial support was provided by Global Brain Health Institute. R. Raman reports financial support was provided by National Institutes of Health. A. Boxer reports financial support was provided by National Institutes of Health. A. Boxer reports financial support was provided by Rainwater Charitable Foundation. A. Boxer reports financial support was provided by Alzheimer’s Association. A. Boxer reports financial support was provided by Association for Frontotemporal Degeneration. A. Boxer reports financial support was provided by GHR Foundation. A. Boxer reports financial support was provided by Alzheimer’s Drug Discovery Foundation. ML. Gorno-Tempini reports financial support was provided by National Institute on Deafness and Other Communication Disorders. ML. Gorno-Tempini reports financial support was provided by National Institute of Neurological Disorders and Stroke. ML. Gorno-Tempini reports financial support was provided by National Institute on Aging. ML. Gorno-Tempini reports financial support was provided by Charles and Helen Schwab Foundation. Ignacio Illan-Gala reports a relationship with Kern Pharma SL that includes: speaking and lecture fees. Ignacio Illan-Gala reports a relationship with Eli Lilly and Company that includes: speaking and lecture fees. Ignacio Illan-Gala reports a relationship with DaThe authors declare that they have no known competing financial interests or personal relationships that could have appeared to influence the work reported in this paper.

## Appendix Supplementary materials

Image, application 1

Image, application 2
