# Supplementary material for: Potential role of MRI to optimize clinical trial design for progressive supranuclear palsy and corticobasal degeneration
Source: J Prev Alzheimers Dis. 2026 Jan 24;13(3):100486. doi: 10.1016/j.tjpad.2026.100486 (PMC12860716; doi:10.1016/j.tjpad.2026.100486)
Supplement: Supplementary file 1 [file mmc1.docx]

**Supplementary Methods**

**MRI Acquisition.** MRI data for participants in the 4RTNI study were acquired using 3 Tesla scanners at UCSF, UCSD, the University of Toronto, and MGH. At UCSF and MGH, imaging was performed on Siemens Tim Trio systems (Siemens, Iselin, NJ) equipped with 12-channel receiver head coils. Whole-brain structural images were obtained using a volumetric magnetization-prepared rapid gradient-echo (MPRAGE) sequence with the following parameters: TR = 2300 ms, TE = 2.98 ms, TI = 900 ms, flip angle = 9°, in-plane resolution = 1 x 1 mm, and slice thickness = 1 mm. At UCSD, scans were acquired on a GE Discovery MR750 system (GE, Milwaukee, WI) with a 32-channel head coil, while at the University of Toronto, a GE Signa HDx scanner with an 8-channel receiver head coil was used. Both UCSD and UToronto employed sagittal 3D inversion-recovery spoiled gradient echo (IR-SPGR) sequences: UCSD parameters were TR = 7.1 ms, TE = 3.00 ms, TI = 400 ms, flip angle = 11°, in-plane resolution = 1 x 1 mm, and slice thickness = 1.2 mm; UToronto parameters were TR = 7.0 ms, TE = 2.80 ms, TI = 400 ms, flip angle = 11°, in-plane resolution = 1 x 1 mm, and slice thickness = 1.2 mm.

For the DAV trial, MRI data were collected using forty-eight 1.5T or 3T scanners with different models but harmonized sequences based on protocols developed by the Mayo Clinic’s Aging and Dementia Imaging Research Laboratory (Rochester, MN, USA), as previously described. All T1-weighted images were acquired using either MPRAGE, coronal IR-SPGR, or sagittal IR-SPGR sequences.

**Supplementary Figure 1. Flowchart of patient selection**


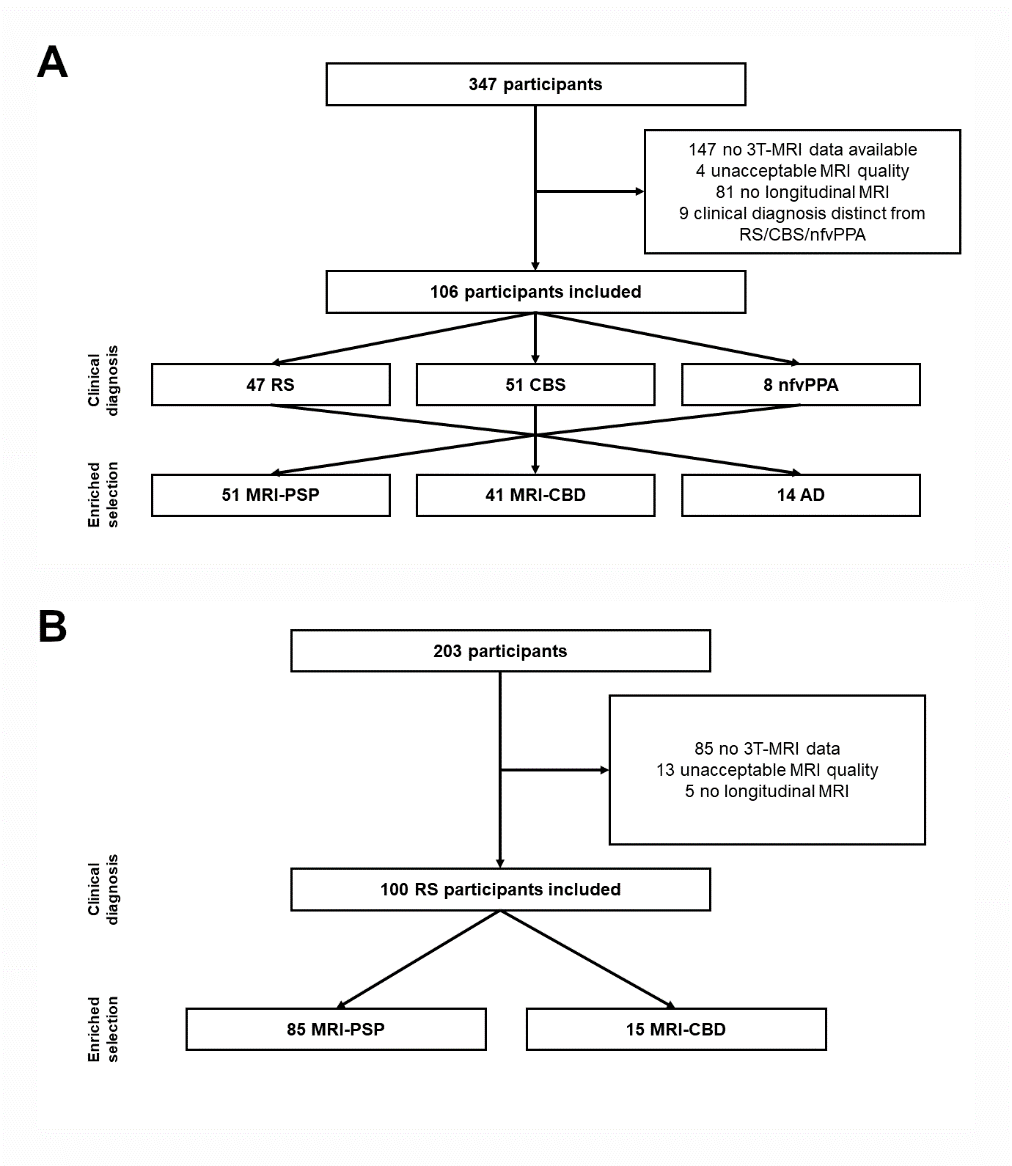


**Footnote:** Flowchart of patient selection for the 4RTNI (A) and Davunetide trial (B) cohorts.

**Abbreviations:** 3T-MRI, 3 Tesla-Magnetic Resonance Imaging; AD, Alzheimer’s Disease; CBS, corticobasal syndrome; CBD, corticobasal degeneration; nfvPPA, Non-fluent variant primary progressive aphasia; PSP, progressive supranuclear palsy; RS, Richardson’s syndrome.

**Supplementary Figure 2. Predicted probability of PSP and optimal cut-off.**

**
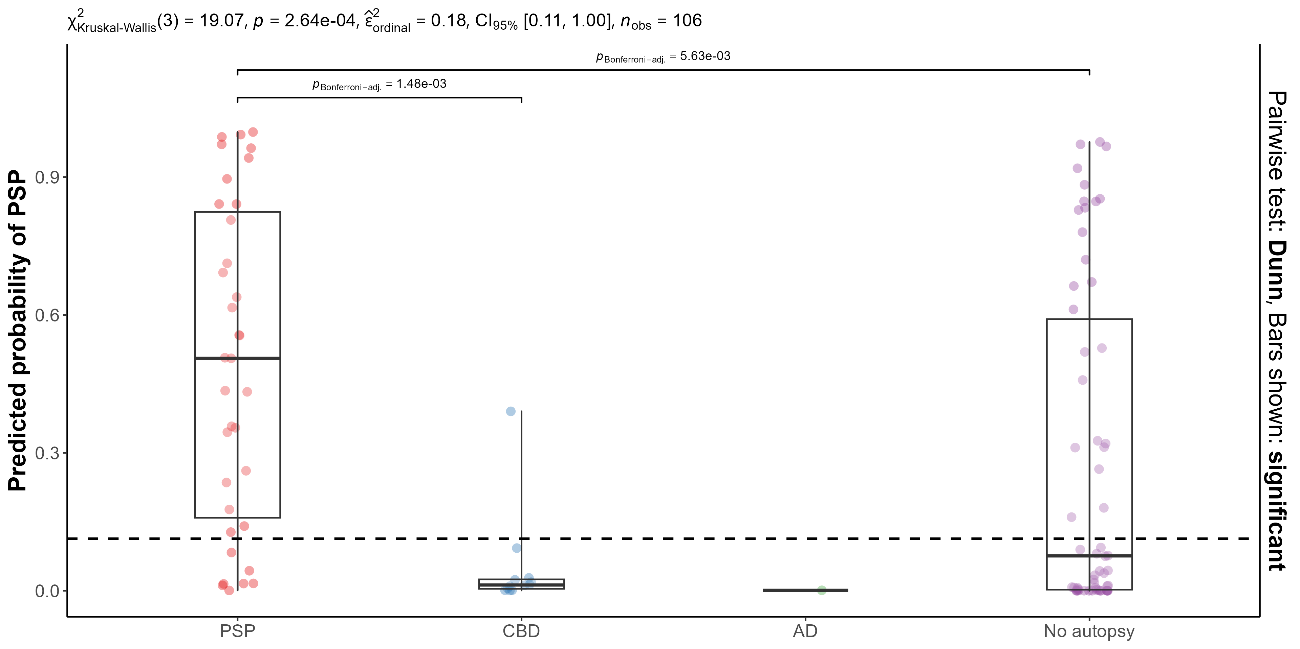
**

**Footnotes:** The panel shows the predicted probability of PSP vs CBD derived from the MLRM in the pathologically confirmed subsamples and the rest of the 4RTNI cohort. The dashed line showed the optimal cutoff for differentiating PSP vs CBD obtained from the autopsy-confirmed subsample using the *cutpointr* R package with 10,000 bootstrap iterations.

**Abbreviations:** AD, Alzheimer’s disease; CBD, corticobasal degeneration; PSP, progressive supranuclear palsy.

**Supplementary Figure 3. Clinical change according to selection strategy**


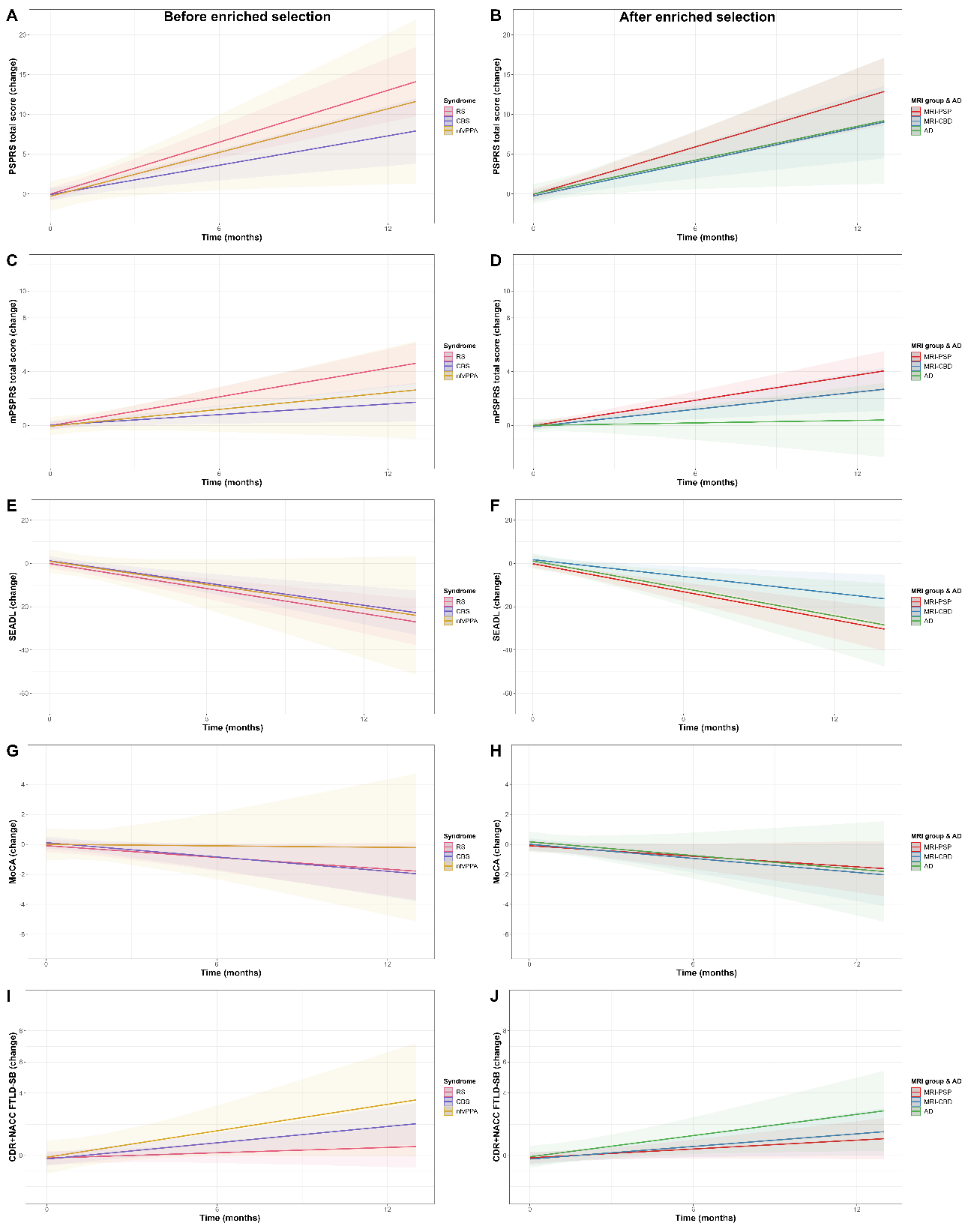


**Footnotes:** Linear mixed-effects models showing the longitudinal change in different scores according to clinical diagnosis only (A) and after the enriched selection using AD biomarkers and MLRM (B).

**Abbreviations:** AD, Alzheimer’s disease; CBD, corticobasal degeneration; CBS, corticobasal syndrome; CDR+NACC FTLD-SB, Clinical Dementia Rating plus National Alzheimer’s Coordinating Center Frontotemporal lobar degeneration sum of boxes; MRI, magnetic resonance imaging; MoCA, Montreal Cognitive Assessment; mPSPRS, modified Progressive supranuclear palsy rating scale; nfvPPA, non-fluent variant of primary progressive aphasia; PSP, progressive supranuclear palsy; RS, Richardson’s syndrome; SEADL, Schwab and England Activities of Daily Living scale.

**Supplementary Figure 4. Patterns of atrophy at 12 months according to clinical diagnosis**


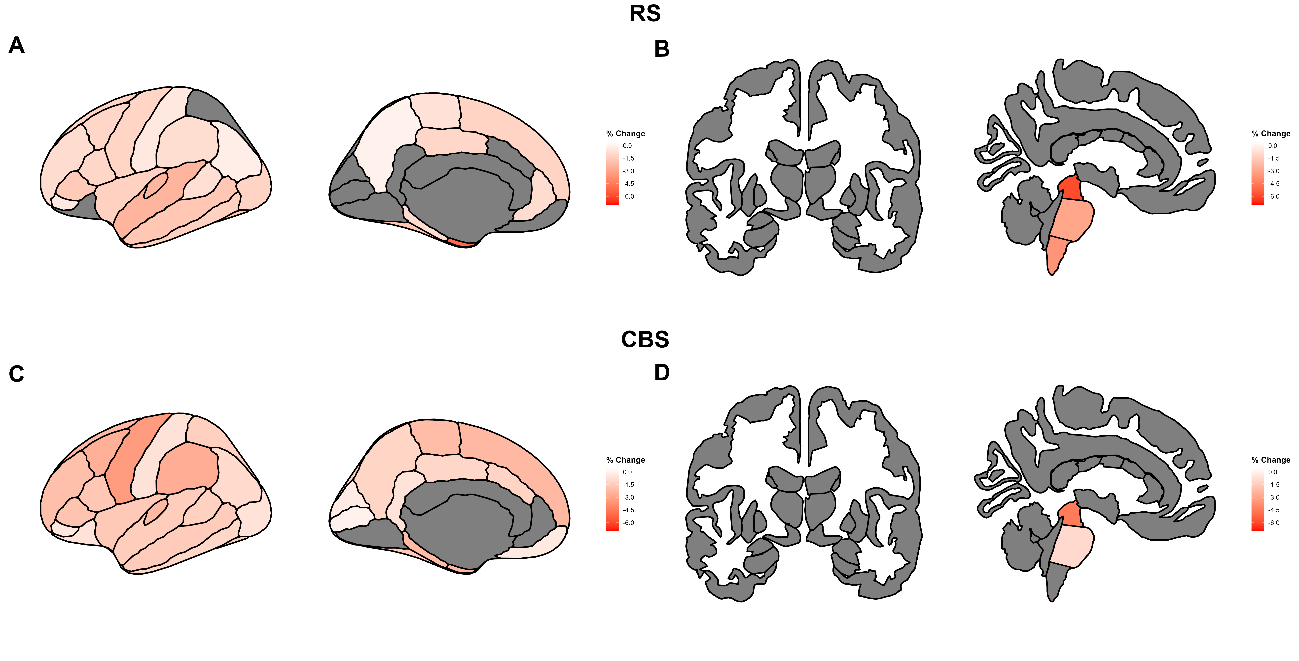


**Footnotes:** Predicted cortical thickness reduction and subcortical volume loss at 12 months in RS (A-B) and CBS (C-D). Values were derived from linear mixed-effects models.

**Abbreviations:** CBS, corticobasal syndrome; MRI, magnetic resonance imaging; RS, Richardson’s syndrome.

**Supplementary Figure 5. Patterns of atrophy at 12 months according to different combinations of clinical diagnosis and predicted pathology**


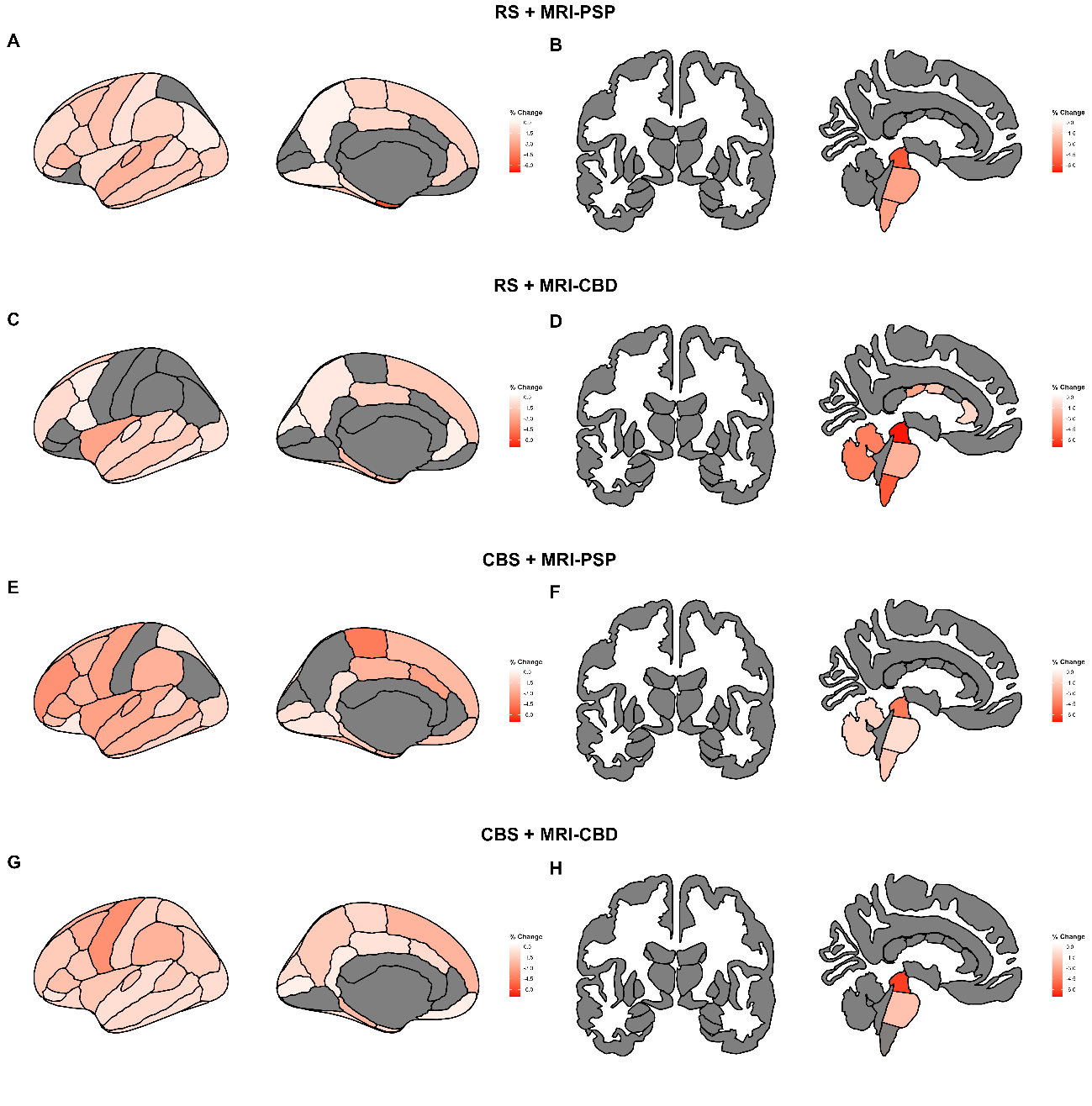


**Footnotes:** Predicted cortical thickness reduction and subcortical volume loss at 12 months in RS + MRI-PSP (A-B), RS + MRI-CBD (C-D), CBS + MRI-PSP (E-F) and CBS + MRI-CBD (G-H). Values were derived from linear mixed-effects models.

**Abbreviations:** CBD, corticobasal degeneration; CBS, corticobasal syndrome; MRI, magnetic resonance imaging; PSP, progressive supranuclear palsy; RS, Richardson’s syndrome.

**Supplementary Figure 6. Patterns of atrophy at 12 months in DAV**


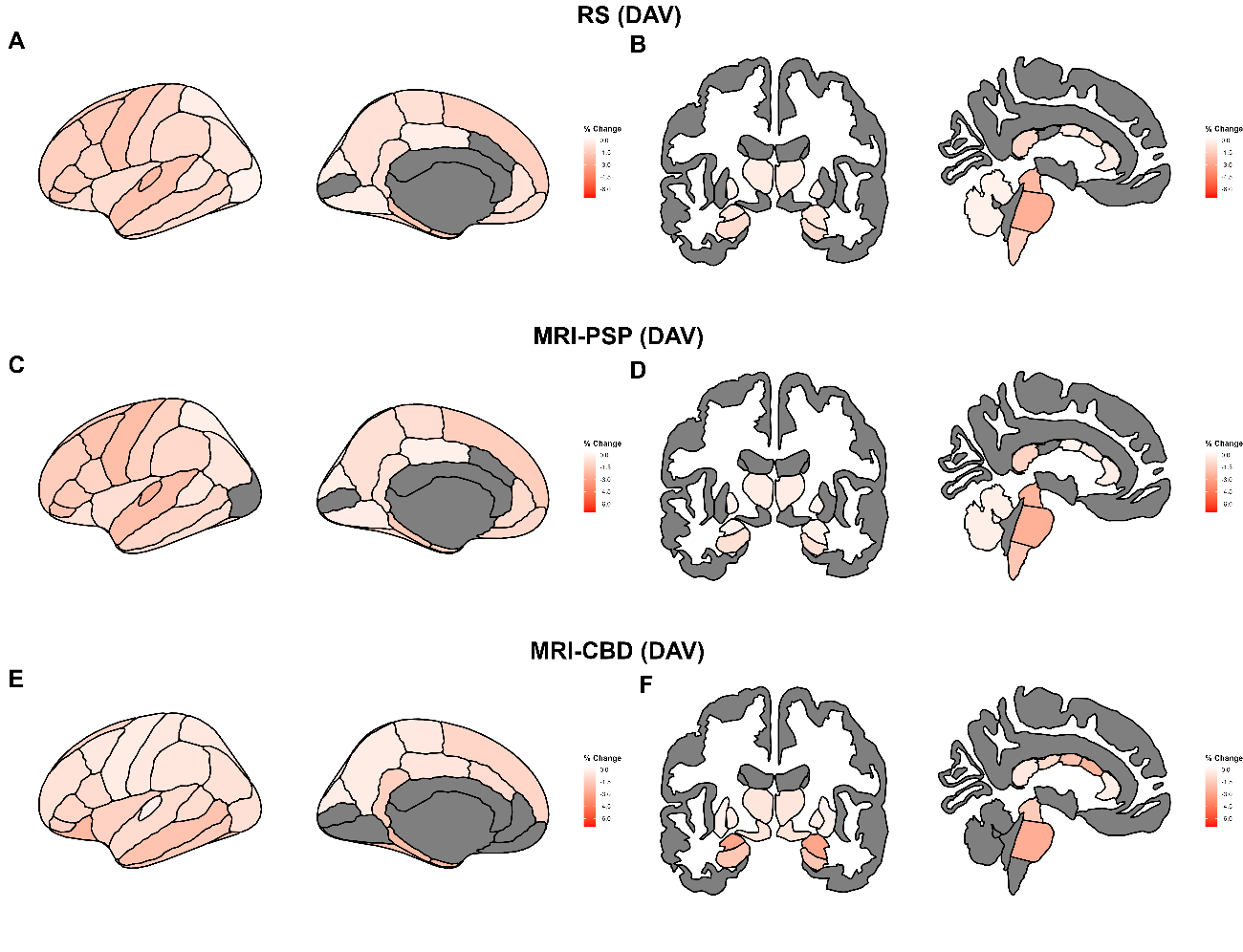


**Footnotes:** Predicted cortical thickness reduction and subcortical volume loss at 12 months in RS (A-B), MRI-PSP (C-D), and MRI-CBD (E-F).

**Abbreviations:** CBS, corticobasal syndrome; DAV, Dabunetide trial cohort; MRI, magnetic resonance imaging; PSP, progressive supranuclear palsy; RS, Richardson’s syndrome.

**Supplementary Figure 7. Correlation between clinical scores and MRI signatures**


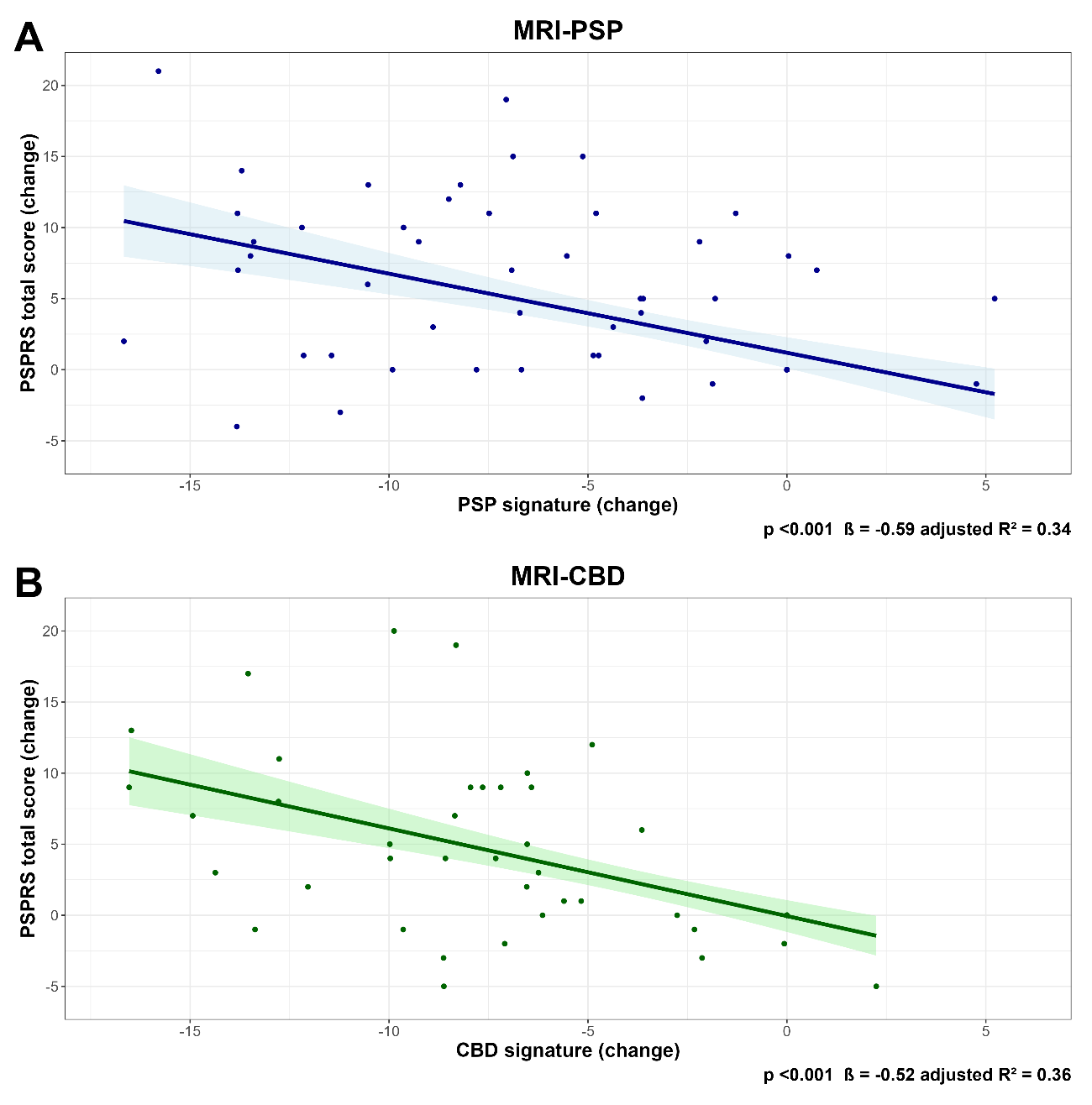


**Footnotes:** Panels show the correlation between clinical score change measured by PSPRS and the most efficient MRI-signatures to detect change for MRI-PSP (A) and MRI-CBD (B) groups. Spearman rho values and p-values are the result of nonparametric Spearman rank order correlations.
**Abbreviations:** MRI, magnetic resonance imaging; CBD, corticobasal degeneration; PSP, progressive supranuclear palsy; PSPRS, progressive supranuclear palsy rating scale.

**Supplementary Figure 8. Longitudinal clinical change for RS and MRI-PSP groups**


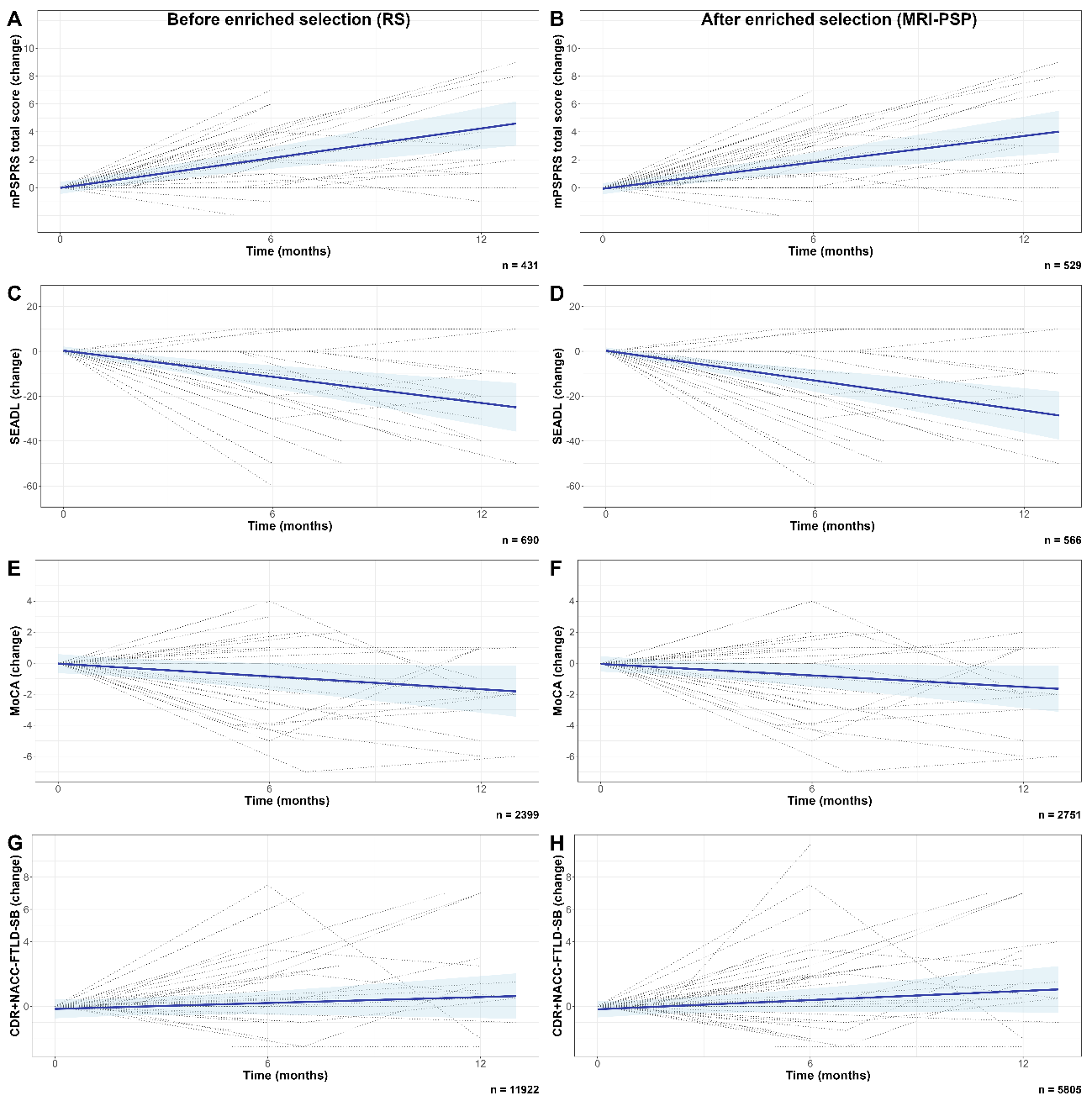


**Footnotes:** Panels show the predicted clinical change as measured by mPSPRS (A,B), SEADL (C,D), MoCA (E,F) and CDR+NACC FTLD-SB (G-H), derived from linear mixed-effects models before enriched selection (inclusion of all participants with RS) and after enriched selection (inclusion of all participants with predicted PSP by MRI [MRI-PSP], regardless of their clinical phenotype). Individual trajectories for each variable are superimposed (dotted lines). Each panel shows the bootstrapped estimated sample size for a clinical trial showing 30% reduction at 12 months with 10% attrition in each variable.

**Abbreviations:** CDR+NACC FTLD-SB, Clinical Dementia Rating plus National Alzheimer’s Coordinating Center Frontotemporal lobar degeneration sum of boxes; MoCA, Montreal cognitive assessment; mPSPRS, modified progressive supranuclear palsy rating scale; PSP, progressive supranuclear palsy; RS, Richardson’s syndrome; SEADL, Schwab and England Activities of Daily Living scale.

**Supplementary Figure 9. Longitudinal clinical change for CBS and MRI-CBD groups**


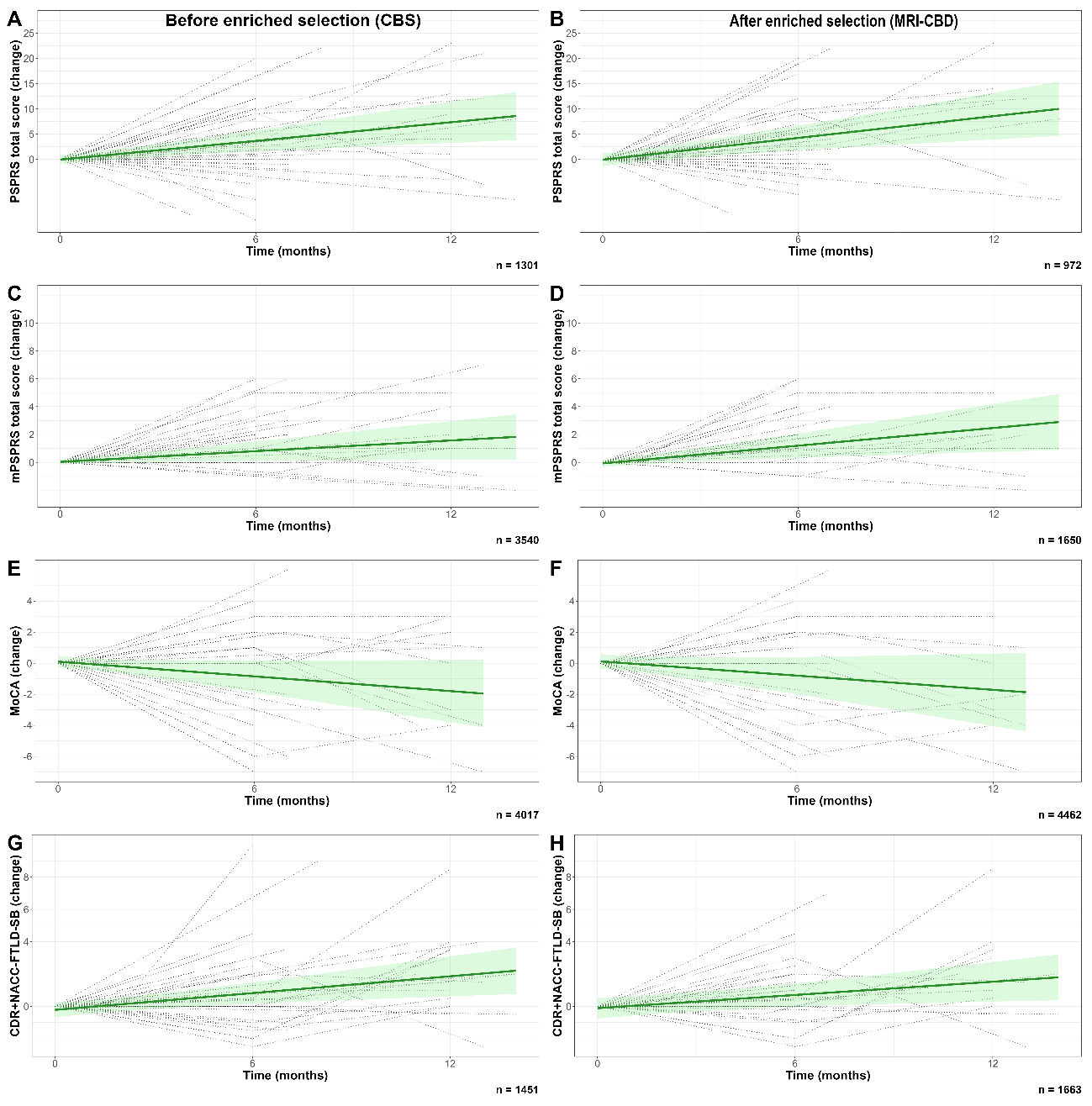


**Footnotes:** Panels show the predicted clinical change as measured by PSPRS (A,B), mPSPRS (C,D), MoCA (E,F) and CDR+NACC FTLD-SB (G-H), derived from linear mixed-effects models before enriched selection (inclusion of all participants with CBS) and after enriched selection (inclusion of all participants with predicted CBD by MRI [MRI-CBD], regardless of their clinical phenotype). Each panel shows the bootstrapped estimated sample size for a clinical trial showing 30% reduction at 12 months with 10% attrition in each variable.

**Abbreviations:** CDR+NACC FTLD-SB, Clinical Dementia Rating plus National Alzheimer’s Coordinating Center Frontotemporal lobar degeneration sum of boxes; MoCA, Montreal cognitive assessment; mPSPRS, modified progressive supranuclear palsy rating scale; PSRS, progressive supranuclear palsy rating scale; SEADL, Schwab and England Activities of Daily Living scale.

**Supplementary Figure 10. Individual trajectories in midbrain percentage change in DAV**


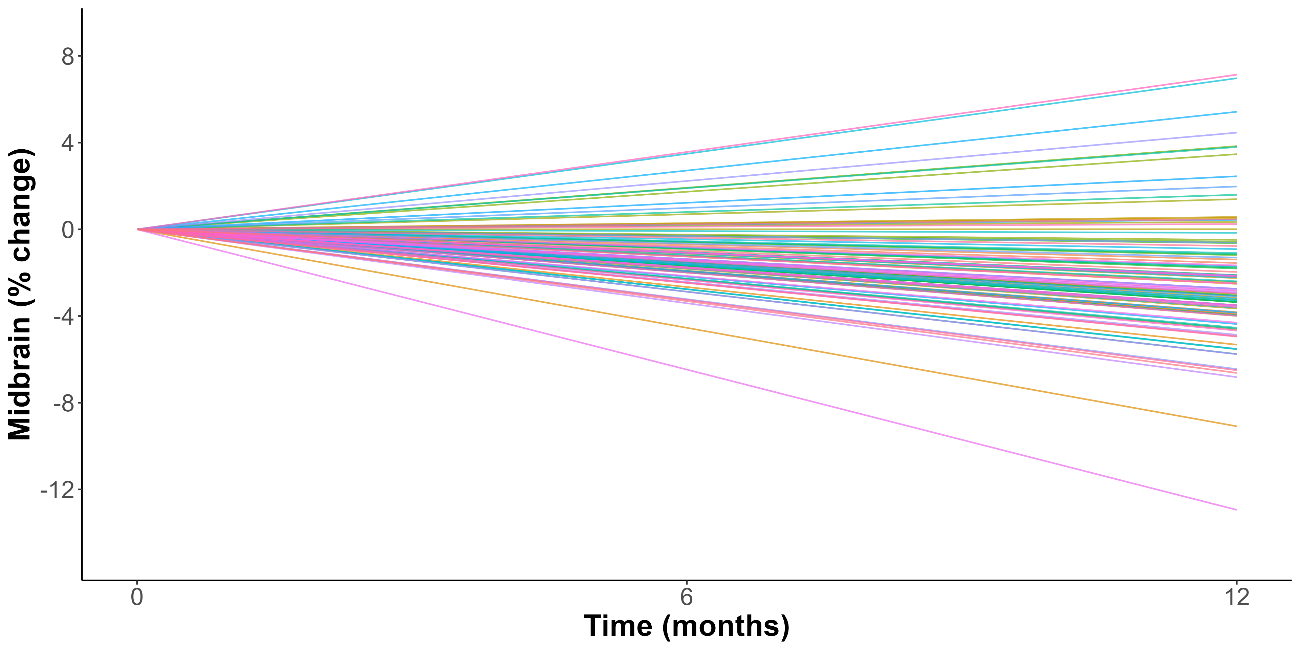


**Abbreviations:** DAV, Davunetide trial cohort.

**Supplementary Table 1. Characteristics of the autopsy subsample**

| **Characteristic** | **All participants, N = 106** | **PSP,**  **N = 35** | **CBD,**  **N = 12** | **AD,**  **N = 1** | **No autopsy, N = 58** | **Adjusted p-value** |
| --- | --- | --- | --- | --- | --- | --- |
| **Age** | 68 (8) | 70 (7) | 65 (7) | 61 (NA) | 67 (9) | >0.9 |
| **Biological Sex** |  | | | | | >0.9 |
| Woman | 53 (50%) | 15 (43%) | 7 (58%) | 0 (0%) | 31 (53%) |  |
| Man | 53 (50%) | 20 (57%) | 5 (42%) | 1 (100%) | 27 (47%) |  |
| **Years of education** | 16.09 (2.97) | 15.03 (3.43) | 17.36 (1.69) | 20.00 (NA) | 16.41 (2.70) | 0.6 |
| **Number of visits** |  | | | | | 0.5 |
| 2 | 23 (22%) | 9 (26%) | 0 (0%) | 0 (0%) | 14 (24%) |  |
| 3 | 63 (59%) | 15 (43%) | 9 (75%) | 1 (100%) | 38 (66%) |  |
| >3 | 20 (19%) | 11 (31%) | 3 (25%) | 0 (0%) | 6 (10%) |  |
| **Clinical phenotype** |  | | | | | <0.001 |
| PSP-RS | 47 (44%) | 32 (91%) | 0 (0%) | 0 (0%) | 15 (26%) |  |
| CBS | 51 (48%) | 2 (6%) | 12 (100%) | 1 (100%) | 36 (62%) |  |
| nfvPPA | 8 (8%) | 1 (3%) | 0 (0%) | 0 (0%) | 7 (12%) |  |
| **PSPRS total score** | 26 (15) | 33 (15) | 24 (14) | 14 (NA) | 22 (14) | 0.13 |
| **modified PSPRS total score** | 4.8 (4.6) | 7.5 (5.4) | 3.0 (3.9) | 2.0 (NA) | 3.6 (3.4) | 0.064 |
| **SEADL total score** | 63 (24) | 60 (25) | 56 (21) | 80 (NA) | 65 (24) | >0.9 |
| **MoCA** | 22.0 (5.0) | 21.1 (4.6) | 22.5 (5.3) | 17.0 (NA) | 22.5 (5.2) | >0.9 |
| **CDR plus NACC FTLD Global score** |  | | | | | >0.9 |
| 0 | 3 (2%) | 2 (5.7%) | 0 (0%) | 0 (0%) | 1 (1%) |  |
| 0.5 | 42 (40%) | 10 (29%) | 4 (33%) | 1 (100%) | 27 (47%) |  |
| 1 | 39 (37%) | 14 (40%) | 6 (50%) | 0 (0%) | 19 (33%) |  |
| 2 | 21 (20%) | 9 (26%) | 1 (8.3%) | 0 (0%) | 11 (19%) |  |
| 3 | 1 (1%) | 0 (0%) | 1 (8.3%) | 0 (0%) | 0 (0%) |  |
| **CDR plus NACC FTLD sum-of-boxes** | 4.7 (3.4) | 4.9 (3.0) | 5.7 (5.1) | 3.5 (NA) | 4.3 (3.2) | >0.9 |
| **Midbrain** | 5,404 (800) | 5,138 (740) | 5,688 (919) | 6,197 (NA) | 5,491 (781) | >0.9 |
| **AD biomarkers** |  | | | | | >0.9 |
| Negative | 63 (75%) | 23 (77%) | 8 (80%) | 0 (0%) | 32 (74%) |  |
| Positive | 21 (25%) | 7 (23%) | 2 (20%) | 1 (100%) | 11 (26%) |  |
| **Amyloid-PET** |  | | | | | >0.9 |
| Negative | 39 (72%) | 6 (67%) | 5 (71%) | 0 (NA%) | 28 (74%) |  |
| Positive | 15 (28%) | 3 (33%) | 2 (29%) | 0 (NA%) | 10 (26%) |  |
| **ptau-217** |  | | | | | >0.9 |
| Negative | 59 (77%) | 22 (76%) | 8 (80%) | 0 (0%) | 29 (78%) |  |
| Positive | 18 (23%) | 7 (24%) | 2 (20%) | 1 (100%) | 8 (22%) |  |
| **Tau-PET** |  | | | | | >0.9 |
| Negative | 38 (83%) | 7 (88%) | 4 (100%) | 0 (NA%) | 27 (79%) |  |
| Positive | 8 (17%) | 1 (13%) | 0 (0%) | 0 (NA%) | 7 (21%) |  |
| **ppPSP** | 0.32 (0.37) | 0.48 (0.35) | 0.03 (0.05) | 0.00 (NA) | 0.30 (0.38) | 0.019 |
| **ppPSPvsCBD** | 0.32 (0.36) | 0.49 (0.35) | 0.05 (0.11) | 0.00 (NA) | 0.28 (0.35) | 0.005 |

**Footnote:** values reported are mean (standard deviation) or n (%). Bonferroni correction for multiple testing was applied in 4RTNI.

**Abbreviations:** AD, Alzheimer’s disease; CBD, corticobasal degeneration; CBS, corticobasal syndrome; CDR+NACC FTLD-GS, Clinical Dementia Rating plus National Alzheimer’s Coordinating Center Frontotemporal lobar degeneration global score; CDR+NACC FTLD-SB, Clinical Dementia Rating plus National Alzheimer’s Coordinating Center Frontotemporal lobar degeneration sum of boxes; MMSE, mini-mental state examination; MoCA, Montreal cognitive assessment; mPSPRS, modified progressive supranuclear palsy rating scale; MRI, magnetic resonance imaging; nfvPPA, non fluent variant of primary progressive aphasia; PET, positron emission tomography; PSP, progressive supranuclear palsy; pp, predicted probability; PSPRS, progressive supranuclear palsy rating scale; RS, Richardson’s syndrome; SEADL,Schwab and England Activities of Daily Living scale.

**Supplementary Table 2. Additional sample characteristics**

|  | **4RTNI** | | | | | **DAV** | | | |
| --- | --- | --- | --- | --- | --- | --- | --- | --- | --- |
| **Characteristic** | **All, N = 106** | **MRI-PSP, N = 51** | **MRI-CBD, N = 41** | **AD, N = 14** | **Adjusted p-value** | **All, N = 100** | **MRI-PSP, N = 85** | **MRI-CBD, N = 15** | **p-value** |
| **Years of education** | 16.09 (2.97) | 15.16 (3.15) | 16.75 (2.69) | 17.57 (1.95) | 0.11 |  | | | |
| **Years of symptoms** | 4.9 (3.4) | 5.1 (3.6) | 5.0 (3.2) | 4.0 (3.5) | >0.9 |  |  |  |  |
| **mPSPRS** | 4.8 (4.6) | 7.3 (4.5) | 3.0 (3.7) | 1.5 (1.5) | **<0.001** |  |  |  |  |
| **MoCA** | 22.0 (5.0) | 22.0 (4.0) | 21.3 (6.0) | 23.8 (5.0) | >0.9 |  |  |  |  |
| **MMSE** |  | | | | | 26.48 (3.06) | 26.58 (2.66) | 25.93 (4.85) | 0.6 |
| **CDR+NACC FTLD-GS** |  | | | | >0.9 |  | | | |
| 0 | 3 (2%) | 3 (6%) | 0 (0%) | 0 (0%) |  |  |  |  |  |
| 0.5 | 42 (40%) | 21 (41%) | 14 (34%) | 7 (50%) |  |  |  |  |  |
| 1 | 39 (37%) | 15 (29%) | 18 (44%) | 6 (43%) |  |  |  |  |  |
| 2 | 21 (20%) | 11 (22%) | 9 (22%) | 1 (7%) |  |  |  |  |  |
| 3 | 1 (1%) | 1 (2%) | 0 (0%) | 0 (0%) |  |  |  |  |  |
| **CDR+NACC FTLD-SB** | 4.7 (3.4) | 4.6 (3.6) | 5.0 (3.4) | 4.0 (2.6) | >0.9 |  |  |  |  |
| **Amyloid-PET** |  | | | | **<0.001** |  |  |  |  |
| Negative | 39 (72%) | 14 (82%) | 25 (100%) | 0 (0%) |  |  |  |  |  |
| Positive | 15 (28%) | 3 (18%) | 0 (0%) | 12 (100%) |  |  |  |  |  |
| **ptau-217** |  | | | | **0.001** |  |  |  |  |
| Negative | 59 (77%) | 29 (85%) | 26 (90%) | 4 (29%) |  |  |  |  |  |
| Positive | 18 (23%) | 5 (15%) | 3 (10%) | 10 (71%) |  |  |  |  |  |
| **Tau-PET** |  | | | | 0.14 |  |  |  |  |
| Negative | 38 (83%) | 14 (93%) | 20 (91%) | 4 (44%) |  |  |  |  |  |
| Positive | 8 (17%) | 1 (6.7%) | 2 (9.1%) | 5 (56%) |  |  |  |  |  |

**Footnote:** values reported are mean (standard deviation) or n (%). Bonferroni correction for multiple testing was applied in 4RTNI.

**Abbreviations:** AD, Alzheimer’s disease; CBD, corticobasal degeneration; CBS, corticobasal syndrome; CDR+NACC FTLD-GS, Clinical Dementia Rating plus National Alzheimer’s Coordinating Center Frontotemporal lobar degeneration global score; CDR+NACC FTLD-SB, Clinical Dementia Rating plus National Alzheimer’s Coordinating Center Frontotemporal lobar degeneration sum of boxes; MMSE, mini-mental state examination; MoCA, Montreal cognitive assessment; mPSPRS, modified progressive supranuclear palsy rating scale; MRI, magnetic resonance imaging; nfvPPA, non fluent variant of primary progressive aphasia; PET, positron emission tomography; PSP, progressive supranuclear palsy; RS, Richardson’s syndrome.

**Supplementary Table 3. Sample characteristics - Clinical diagnosis only**

| **Characteristic** | **All participants, N = 106** | **RS,**  **N = 47** | **CBS,**  **N = 51** | **nfvPPA,**  **N = 8** | **Adjusted p-value** |
| --- | --- | --- | --- | --- | --- |
| **Age** | 68 (8) | 70 (8) | 65 (9) | 70 (7) | 0.7 |
| **Biological Sex** |  | | | | >0.9 |
| Woman | 53 (50%) | 25 (53%) | 27 (53%) | 1 (13%) |  |
| Man | 53 (50%) | 22 (47%) | 24 (47%) | 7 (88%) |  |
| **Years of education** | 16.09 (2.97) | 15.15 (3.27) | 16.74 (2.46) | 17.50 (2.78) | 0.2 |
| **Number of visits** |  | | | | >0.9 |
| 2 | 23 (22%) | 13 (28%) | 8 (16%) | 2 (25%) |  |
| 3 | 63 (59%) | 26 (55%) | 33 (65%) | 4 (50%) |  |
| >3 | 20 (19%) | 8 (17%) | 10 (20%) | 2 (25%) |  |
| **MRI-based classification** |  | | | | **<0.001** |
| CBD | 52 (49%) | 8 (17%) | 37 (73%) | 7 (88%) |  |
| PSP | 54 (51%) | 39 (83%) | 14 (27%) | 1 (13%) |  |
| **Years of symptoms** | 4.9 (3.4) | 5.5 (3.9) | 4.5 (2.9) | 4.1 (3.1) | >0.9 |
| **PSPRS total score** | 26 (15) | 33 (15) | 23 (12) | 8 (5) | **<0.001** |
| **modified PSPRS total score** | 4.8 (4.6) | 7.5 (4.7) | 3.1 (3.3) | 0.6 (0.7) | **<0.001** |
| **SEADL total score** | 63 (24) | 58 (25) | 66 (22) | 76 (19) | >0.9 |
| **MoCA** | 22.0 (5.0) | 21.6 (4.3) | 22.0 (5.5) | 24.2 (5.4) | >0.9 |
| **CDR plus NACC FTLD Global score** |  | | | | >0.9 |
| 0 | 3 (2%) | 3 (6%) | 0 (0%) | 0 (0%) |  |
| 0.5 | 42 (40%) | 14 (30%) | 24 (47%) | 4 (50%) |  |
| 1 | 39 (37%) | 17 (36%) | 20 (39%) | 2 (25%) |  |
| 2 | 21 (20%) | 13 (28%) | 6 (12%) | 2 (25%) |  |
| 3 | 1 (1%) | 0 (0%) | 1 (2%) | 0 (0%) |  |
| **CDR plus NACC FTLD sum-of-boxes** | 4.7 (3.4) | 5.0 (3.1) | 4.4 (3.6) | 4.4 (4.1) | >0.9 |
| **Midbrain** | 5,404 (800) | 5,063 (672) | 5,574 (797) | 6,321 (377) | **<0.001** |
| **A+** |  | | | | >0.9 |
| Negative | 63 (75%) | 28 (80%) | 30 (71%) | 5 (71%) |  |
| Positive | 21 (25%) | 7 (20%) | 12 (29%) | 2 (29%) |  |
| **Amyloid-PET** |  | | | | >0.9 |
| Negative | 39 (72%) | 11 (79%) | 23 (70%) | 5 (71%) |  |
| Positive | 15 (28%) | 3 (21%) | 10 (30%) | 2 (29%) |  |
| **ptau-217** |  | | | | >0.9 |
| Negative | 59 (77%) | 24 (77%) | 28 (72%) | 7 (100%) |  |
| Positive | 18 (23%) | 7 (23%) | 11 (28%) | 0 (0%) |  |
| **Tau-PET** |  | | | | >0.9 |
| Negative | 38 (83%) | 12 (86%) | 19 (76%) | 7 (100%) |  |
| Positive | 8 (17%) | 2 (14%) | 6 (24%) | 0 (0%) |  |
| **Stability of MRI-based classification** |  | | | | >0.9 |
| CBD to PSP | 14 (13%) | 4 (8.5%) | 10 (20%) | 0 (0%) |  |
| Stable | 92 (87%) | 43 (91%) | 41 (80%) | 8 (100%) |  |

**Footnote:** values reported are mean (standard deviation) or n (%).

**Abbreviations:** AD, Alzheimer’s disease; CBD, corticobasal degeneration; CBS, corticobasal syndrome; CDR+NACC FTLD-GS, Clinical Dementia Rating plus National Alzheimer’s Coordinating Center Frontotemporal lobar degeneration global score; CDR+NACC FTLD-SB, Clinical Dementia Rating plus National Alzheimer’s Coordinating Center Frontotemporal lobar degeneration sum of boxes; MMSE, mini-mental state examination; MoCA, Montreal cognitive assessment; mPSPRS, modified progressive supranuclear palsy rating scale; MRI, magnetic resonance imaging; nfvPPA, non fluent variant of primary progressive aphasia; PET, positron emission tomography; PSP, progressive supranuclear palsy; PSPRS, progressive supranuclear palsy rating scale; RS, Richardson’s syndrome; SEADL,Schwab and England Activities of Daily Living scale.

**Supplementary Table 4. Linear mixed effect model for PSPRS**

| **Before enriched selection** | | | | | | **After enriched selection** | | | | | |
| --- | --- | --- | --- | --- | --- | --- | --- | --- | --- | --- | --- |
|  | **Estimate** | **Std. Error** | **df** | **t value** | **p-value** |  | **Estimate** | **Std. Error** | **df** | **t value** | **p-value** |
| **Intercept** | 0,258 | 0,678 | 126,561 | 0,38 | 0,705 | **Intercept** | 0,196 | 0,65 | 129,297 | 0,301 | 0,764 |
| **Time (months)** | 1,089 | 0,173 | 104,851 | 6,282 | **<0.001** | **Time (months)** | 0,995 | 0,17 | 105,445 | 5,854 | **<0.001** |
| **Sex (male)** | -0,109 | 0,432 | 132,789 | -0,253 | 0,801 | **Sex (male)** | -0,058 | 0,431 | 132,212 | -0,135 | 0,893 |
| **Basal PSPRS** | -0,011 | 0,017 | 131,747 | -0,644 | 0,521 | **Basal PSPRS** | -0,009 | 0,017 | 134,512 | -0,536 | 0,593 |
| **Group (CBS)** | -0,059 | 0,49 | 116,406 | -0,12 | 0,904 | **Group (MRI-CBD)** | -0,175 | 0,512 | 117,893 | -0,342 | 0,733 |
| **Group (nfvPPA)** | -0,263 | 0,989 | 116,95 | -0,266 | 0,791 | **Group (AD)** | 0,065 | 0,757 | 117,327 | 0,085 | 0,932 |
| **Time:Group (CBS)** | -0,473 | 0,239 | 104,882 | -1,982 | **0,050** | **Time:Group (MRI-CBD)** | -0,28 | 0,25 | 106,675 | -1,121 | 0,265 |
| **Time:Group (nfvPPA)** | -0,172 | 0,452 | 118,922 | -0,38 | 0,704 | **Time:Group (AD)** | -0,286 | 0,36 | 102,966 | -0,794 | 0,429 |

**Footnote:**  Linear mixed-effects models showing the longitudinal change in PSPRS according to clinical diagnosis only and after the enriched selection using AD biomarkers and multinomial logistic regression model.

**Abbreviations:** AD, Alzheimer’s disease; CBD, corticobasal degeneration; CBS, corticobasal syndrome;  df, degrees of freedom; MRI, magnetic resonance imaging; nfvPPA, non fluent variant of primary progressive aphasia; PSP, progressive supranuclear palsy; PSPRS, progressive supranuclear palsy rating scale, RS, Richardson’s syndrome.

**Supplementary Table 5. Linear mixed effect model for midbrain volume**

| **Before enriched selection** | | | | | | **After enriched selection** | | | | | |
| --- | --- | --- | --- | --- | --- | --- | --- | --- | --- | --- | --- |
|  | **Estimate** | **Std. Error** | **df** | **t value** | **p-value** |  | **Estimate** | **Std. Error** | **df** | **t value** | **p-value** |
| **Intercept** | 0,915 | 0,866 | 169,869 | 1,056 | 0,293 | **Intercept** | 0,296 | 1,055 | 177,711 | 0,28 | 0,779 |
| **Time (months)** | -0,485 | 0,061 | 124,551 | -7,89 | **<0.001** | **Time (months)** | -0,44 | 0,056 | 125,309 | -7,896 | **<0.001** |
| **Sex (male)** | -0,039 | 0,261 | 172,328 | -0,148 | 0,883 | **Sex (male** | -0,096 | 0,263 | 177,02 | -0,364 | 0,716 |
| **Basal midbrain volume** | 0 | 0 | 172,114 | -1,087 | 0,279 | **Basal midbrain volume** | 0 | 0 | 180,211 | -0,228 | 0,82 |
| **Group (CBS)** | -0,032 | 0,262 | 136,356 | -0,12 | 0,905 | **Group (MRI-CBD)** | -0,178 | 0,346 | 150,78 | -0,513 | 0,609 |
| **Group (nfvPPA)** | 0,502 | 0,522 | 133,463 | 0,961 | 0,338 | **Group (AD)** | -0,227 | 0,438 | 139,744 | -0,517 | 0,606 |
| **Time:Group (CBS)** | 0,123 | 0,086 | 124,668 | 1,426 | 0,156 | **Time:Group (MRI-CBD)** | -0,099 | 0,085 | 126,201 | -1,166 | 0,246 |
| **Time:Group (nfvPPA)** | -0,004 | 0,176 | 166,864 | -0,024 | 0,981 | **Time:Group (AD)** | 0,379 | 0,12 | 124,153 | 3,149 | **0,002** |

**Footnote:**  Linear mixed-effects models showing the longitudinal change in midbrain volume according to clinical diagnosis only and after the enriched selection using AD biomarkers and multinomial logistic regression model.

**Abbreviations:** AD, Alzheimer’s disease; CBD, corticobasal degeneration; CBS, corticobasal syndrome; df, degrees of freedom;  MRI, magnetic resonance imaging; nfvPPA, non fluent variant of primary progressive aphasia.

**Supplementary Table 6. Linear mixed effect model for additional clinical variables**

| **Before enriched selection** | | | | | | | **After enriched selection** | | | | | |
| --- | --- | --- | --- | --- | --- | --- | --- | --- | --- | --- | --- | --- |
| **mPSPRS** | | | | | | | | | | | | |
|  | **Estimate** | **SE** | **df** | **t value** | **p-value** |  | | **Estimate** | **SE** | **df** | **t value** | **p-value** |
| **Intercept** | 0,047 | 0,217 | 122,12 | 0,215 | 0,83 | **Intercept** | | 0,072 | 0,203 | 124,323 | 0,357 | 0,722 |
| **Time (months)** | 0,357 | 0,061 | 101,081 | 5,854 | **<0.001** | **Time (months)** | | 0,313 | 0,06 | 102,233 | 5,258 | **<0.001** |
| **Sex (male)** | -0,07 | 0,164 | 130,616 | -0,426 | 0,671 | **Sex (male)** | | -0,046 | 0,163 | 130,532 | -0,282 | 0,778 |
| **Basal mPSPRS** | -0,015 | 0,021 | 131,646 | -0,685 | 0,495 | **Basal mPSPRS** | | -0,015 | 0,021 | 134,239 | -0,716 | 0,475 |
| **Group (CBS)** | 0,034 | 0,197 | 113,458 | 0,171 | 0,864 | **Group (MRI-CBD)** | | -0,064 | 0,201 | 114,668 | -0,318 | 0,751 |
| **Group (nfvPPA)** | -0,034 | 0,368 | 111,404 | -0,093 | 0,926 | **Group (AD)** | | 0,024 | 0,29 | 113,137 | 0,082 | 0,934 |
| **Time*Group (CBS)** | -0,226 | 0,084 | 101,088 | -2,683 | **0,009** | **Time*Group (MRI-CBD)** | | -0,099 | 0,088 | 103,655 | -1,131 | 0,261 |
| **Time*Group (nfvPPA)** | -0,15 | 0,16 | 117,981 | -0,938 | 0,35 | **Time*Group (AD)** | | -0,282 | 0,126 | 99,347 | -2,239 | **0,027** |
| **SEADL** | | | | | | | | | | | | |
|  | **Estimate** | **SE** | **df** | **t value** | **p-value** |  | | **Estimate** | **SE** | **df** | **t value** | **p-value** |
| **Intercept** | 1,743 | 1,84 | 127,044 | 0,947 | 0,345 | **Intercept** | | 1,672 | 1,81 | 128,658 | 0,924 | 0,357 |
| **Time (months)** | -1,928 | 0,401 | 101,14 | -4,809 | **<0.001** | **Time (months)** | | -2,16 | 0,38 | 103,347 | -5,679 | **<0.001** |
| **Basal mPSPRS** | 0,313 | 1,254 | 133,005 | 0,249 | 0,804 | **Basal mPSPRS** | | -0,157 | 1,238 | 133,735 | -0,127 | 0,899 |
| **Sex (male)** | -0,028 | 0,027 | 133,565 | -1,052 | 0,295 | **Sex (male)** | | -0,028 | 0,026 | 136,488 | -1,05 | 0,295 |
| **Group (CBS)** | 1,234 | 1,324 | 106,31 | 0,932 | 0,354 | **Group (MRI-CBD)** | | 1,901 | 1,383 | 107,92 | 1,375 | 0,172 |
| **Group (nfvPPA)** | 1,03 | 2,747 | 105,76 | 0,375 | 0,709 | **Group (AD)** | | 1,311 | 2,049 | 106,986 | 0,64 | 0,524 |
| **Time*Group (CBS)** | 0,216 | 0,555 | 101,427 | 0,389 | 0,698 | **Time*Group (MRI-CBD)** | | 0,869 | 0,566 | 105,719 | 1,536 | 0,127 |
| **Time*Group (nfvPPA)** | 0,142 | 1,123 | 127,122 | 0,126 | 0,9 | **Time*Group (AD)** | | 0,049 | 0,82 | 99,35 | 0,06 | 0,952 |
| **MoCA** | | | | | | | | | | | | |
|  | **Estimate** | **SE** | **df** | **t value** | **p-value** |  | | **Estimate** | **SE** | **df** | **t value** | **p-value** |
| **Intercept** | -0,361 | 0,562 | 124,594 | -0,641 | 0,522 | **Intercept** | | -0,322 | 0,563 | 124,588 | -0,571 | 0,569 |
| **Time (months)** | -0,131 | 0,077 | 88,165 | -1,691 | 0,094 | **Time (months)** | | -0,117 | 0,075 | 90,961 | -1,56 | 0,122 |
| **Sex (male)** | -0,083 | 0,238 | 128,375 | -0,35 | 0,727 | **Sex (male)** | | -0,105 | 0,238 | 128,3 | -0,442 | 0,659 |
| **Basal mPSPRS** | 0,013 | 0,024 | 127,247 | 0,532 | 0,596 | **Basal mPSPRS** | | 0,013 | 0,024 | 126,337 | 0,524 | 0,601 |
| **Group (CBS)** | 0,202 | 0,256 | 103,584 | 0,788 | 0,432 | **Group (MRI-CBD)** | | 0,115 | 0,271 | 104,502 | 0,423 | 0,673 |
| **Group (nfvPPA)** | 0,085 | 0,55 | 103,39 | 0,155 | 0,877 | **Group (AD)** | | 0,281 | 0,39 | 103,519 | 0,72 | 0,473 |
| **Time*Group (CBS)** | -0,029 | 0,108 | 91,238 | -0,267 | 0,79 | **Time*Group (MRI-CBD)** | | -0,039 | 0,113 | 92,582 | -0,347 | 0,729 |
| **Time*Group (nfvPPA)** | 0,114 | 0,219 | 114,386 | 0,521 | 0,603 | **Time*Group (AD)** | | -0,035 | 0,157 | 96,116 | -0,225 | 0,823 |
| **CDR + NACC FTLD-SB** | | | | | | | | | | | | |
|  | **Estimate** | **SE** | **df** | **t value** | **p-value** |  | | **Estimate** | **SE** | **df** | **t value** | **p-value** |
| **Intercept** | 0,041 | 0,279 | 144,525 | 0,148 | 0,882 | **Intercept** | | 0,041 | 0,258 | 143,701 | 0,157 | 0,875 |
| **Time (months)** | 0,057 | 0,057 | 109,571 | 0,992 | 0,323 | **Time (months)** | | 0,095 | 0,056 | 112,762 | 1,685 | 0,095 |
| **Sex (male)** | 0,023 | 0,238 | 175,729 | 0,095 | 0,924 | **Sex (male)** | | 0,049 | 0,238 | 174,486 | 0,204 | 0,839 |
| **Basal mPSPRS** | -0,045 | 0,036 | 170,304 | -1,264 | 0,208 | **Basal mPSPRS** | | -0,046 | 0,036 | 168,72 | -1,28 | 0,202 |
| **Group (CBS)** | -0,062 | 0,269 | 118,557 | -0,229 | 0,819 | **Group (MRI-CBD)** | | -0,09 | 0,282 | 119,248 | -0,319 | 0,75 |
| **Group (nfvPPA)** | 0,055 | 0,542 | 115,239 | 0,101 | 0,92 | **Group (AD)** | | 0,056 | 0,409 | 118,168 | 0,138 | 0,89 |
| **Time*Group (CBS)** | 0,117 | 0,08 | 109,871 | 1,458 | 0,148 | **Time*Group (MRI-CBD)** | | 0,041 | 0,085 | 114,062 | 0,479 | 0,633 |
| **Time*Group (nfvPPA)** | 0,226 | 0,168 | 178,108 | 1,344 | 0,181 | **Time*Group (AD)** | | 0,132 | 0,122 | 110,862 | 1,081 | 0,282 |

**Footnote:**  Linear mixed-effects models showing the longitudinal change in clinical scores according to clinical diagnosis only and after the enriched selection using AD biomarkers and multinomial logistic regression model.

**Abbreviations:** AD, Alzheimer’s disease; CBD, corticobasal degeneration; CBS, corticobasal syndrome; CDR+NACC FTLD-SB, Clinical Dementia Rating plus National Alzheimer’s Coordinating Center Frontotemporal lobar degeneration sum of boxes; df, degrees of freedom; mPSPRS, modified progressive supranuclear palsy rating scale; MoCA, Montreal cognitive assessment; MRI, magnetic resonance imaging; nfvPPA, non fluent variant of primary progressive aphasia; PSP, progressive supranuclear palsy; RS, Richardson’s syndrome; SE, standard error; SEADL,Schwab and England Activities of Daily Living scale

**Supplementary Table 7. Linear models for PSPRS change and midbrain atrophy in the DAV cohort**

| **PSPRS** | | | | |
| --- | --- | --- | --- | --- |
|  | **Estimate** | **SE** | **t value** | **p-value** |
| **Intercept** | 2.541 | 1.915 | 1.327 | 0.186 |
| **Time (months)** | 0.960 | 0.092 | 10.462 | **<0.001** |
| **Sex (female)** | 0.179 | 1.025 | 0.175 | 0.861 |
| **Basal PSPRS** | -0.066 | 0.043 | -1.546 | 0.124 |
| **Group (MRI-CBD)** | -0.700 | 2.043 | -0.343 | 0.732 |
| **Time:Group (MRI-CBD)** | 0.084 | 0.235 | 0.358 | 0.721 |
| **Midbrain** | | | | |
|  | **Estimate** | **SE** | **t value** | **p-value** |
| **Intercept** | 1.281 | 1.422 | 0.900 | 0.369 |
| **Time (months)** | -0.201 | 0.027 | -7.362 | **<0.001** |
| **Sex (female)** | -0.385 | 0.381 | -1.010 | 0.314 |
| **Basal midbrain volume** | 0 | 0 | -0.744 | 0.458 |
| **Group (MRI-CBD)** | 0.285 | 0.655 | 0.435 | 0.664 |
| **Time:Group (MRI-CBD)** | 0.076 | 0.071 | 1.072 | 0.285 |

**Footnote:** Linear models showing the longitudinal change in PSPRS and midbrain volume after the enriched selection using the multinomial logistic regression model. MRI-PSP was used as the reference group.

**Abbreviations:** CBD, corticobasal degeneration; DAV, Davunetide trial cohort; PSPRS, progressive supranuclear palsy rating scale; MRI, magnetic resonance imaging; PSP, progressive supranuclear palsy; RS, Richardson’s syndrome; SE, standard error

**Supplementary Table 8. Predicted atrophy at 12 months - MRI-PSP**

|  | **ROI** | **Predicted atrophy** | **95% CI** | **SE** |
| --- | --- | --- | --- | --- |
| 1 | Superior cerebellar peduncle | -7.944 | -12.743 -- -3.145 | 2.422 |
| 2 | Midbrain | -5.215 | -6.407 -- -4.022 | 0.602 |
| 3 | Entorhinal | -4.959 | -7.385 -- -2.534 | 1.224 |
| 4 | Transverse temporal | -2.518 | -4.371 -- -0.664 | 0.935 |
| 5 | Pons | -2.398 | -3.194 -- -1.603 | 0.401 |
| 6 | Superior temporal | -2.391 | -3.404 -- -1.378 | 0.511 |
| 7 | Medulla | -2.307 | -5.242 -- 0.627 | 1.481 |
| 8 | Pars triangularis | -2.176 | -3.481 -- -0.871 | 0.659 |
| 9 | Paracentral | -1.961 | -3.958 -- 0.037 | 1.008 |
| 10 | Precentral | -1.931 | -3.537 -- -0.324 | 0.811 |
| 11 | Fusiform | -1.914 | -3.208 -- -0.621 | 0.653 |
| 12 | Middle temporal | -1.871 | -2.816 -- -0.925 | 0.477 |
| 13 | Caudal middle frontal | -1.831 | -2.934 -- -0.728 | 0.557 |
| 14 | Frontal pole | -1.724 | -4.646 -- 1.198 | 1.475 |
| 15 | Pars opercularis | -1.544 | -2.512 -- -0.576 | 0.488 |
| 16 | Rostral middle frontal | -1.506 | -2.925 -- -0.086 | 0.716 |
| 17 | Posterior cingulate | -1.449 | -2.892 -- -0.005 | 0.729 |
| 18 | Superior frontal | -1.422 | -2.49 -- -0.354 | 0.539 |
| 19 | Insula | -1.4 | -2.834 -- 0.034 | 0.724 |
| 20 | Temporal pole | -1.376 | -3.835 -- 1.084 | 1.241 |
| 21 | Inferior temporal | -1.372 | -2.448 -- -0.295 | 0.543 |
| 22 | Supramarginal | -1.364 | -2.537 -- -0.19 | 0.592 |
| 23 | Lateral occipital | -1.256 | -2.364 -- -0.147 | 0.56 |
| 24 | Pars orbitalis | -1.033 | -2.407 -- 0.34 | 0.693 |
| 25 | Parahippocampal | -0.876 | -2.568 -- 0.815 | 0.853 |
| 26 | Banks of the superior temporal sulcus | -0.865 | -2.056 -- 0.326 | 0.601 |
| 27 | Caudal anterior cingulate | -0.861 | -2.835 -- 1.114 | 0.996 |
| 28 | Rostral anterior cingulate | -0.732 | -2.694 -- 1.23 | 0.99 |
| 29 | Postcentral | -0.17 | -1.501 -- 1.16 | 0.671 |
| 30 | Lingual | -0.142 | -1.372 -- 1.089 | 0.621 |

**Footnote:**  Predicted cortical thickness reduction and subcortical volume loss at 12 months in MRI-PSP. Values were derived from linear mixed-effects models

**Abbreviations:** CI, confidence interval; PSP, progressive supranuclear palsy; ROI, region of interest; SE, standard error.

**Supplementary Table 9. Predicted atrophy at 12 months - MRI-CBD**

|  | **ROI** | **Predicted atrophy** | **95% CI** | **SE** |
| --- | --- | --- | --- | --- |
| 1 | Midbrain | -6.543 | -7.839 -- -5.247 | 0.652 |
| 2 | Superior cerebellar peduncle | -3.58 | -7.647 -- 0.487 | 2.046 |
| 3 | Parahippocampal | -2.369 | -4.145 -- -0.592 | 0.894 |
| 4 | Pons | -2.245 | -3.076 -- -1.414 | 0.418 |
| 5 | Supramarginal | -2.178 | -3.862 -- -0.495 | 0.847 |
| 6 | Superior frontal | -2.124 | -3.377 -- -0.871 | 0.63 |
| 7 | Precentral | -1.933 | -5.581 -- 1.716 | 1.835 |
| 8 | Insula | -1.931 | -3.751 -- -0.11 | 0.916 |
| 9 | Frontal pole | -1.862 | -4.179 -- 0.456 | 1.166 |
| 10 | Caudal middle frontal | -1.645 | -3.903 -- 0.614 | 1.136 |
| 11 | Rostral middle frontal | -1.642 | -3.446 -- 0.163 | 0.908 |
| 12 | Banks of the superior temporal sulcus | -1.628 | -2.983 -- -0.273 | 0.682 |
| 13 | Postcentral | -1.612 | -4.138 -- 0.914 | 1.271 |
| 14 | Pars opercularis | -1.598 | -3.37 -- 0.174 | 0.892 |
| 15 | Pars triangularis | -1.567 | -3.217 -- 0.084 | 0.831 |
| 16 | Inferior parietal | -1.493 | -2.735 -- -0.25 | 0.625 |
| 17 | Transverse temporal | -1.433 | -3.383 -- 0.518 | 0.981 |
| 18 | Middle temporal | -1.362 | -2.192 -- -0.533 | 0.417 |
| 19 | Precuneus | -1.27 | -2.775 -- 0.234 | 0.757 |
| 20 | Entorhinal | -1.21 | -3.34 -- 0.92 | 1.072 |
| 21 | Superior temporal | -1.117 | -2.134 -- -0.1 | 0.511 |
| 22 | Superior parietal | -1.028 | -2.643 -- 0.586 | 0.812 |
| 23 | Posterior cingulate | -0.895 | -2.416 -- 0.627 | 0.765 |
| 24 | Inferior temporal | -0.892 | -1.819 -- 0.035 | 0.466 |
| 25 | Cerebellum cortex | -0.833 | -3.25 -- 1.584 | 1.216 |
| 26 | Fusiform | -0.695 | -2.139 -- 0.748 | 0.726 |
| 27 | Lateral occipital | -0.671 | -1.77 -- 0.427 | 0.553 |
| 28 | Cuneus | -0.632 | -2.202 -- 0.938 | 0.79 |
| 29 | Pars orbitalis | -0.509 | -2.397 -- 1.379 | 0.95 |
| 30 | Lateral orbitofrontal | -0.456 | -2.277 -- 1.365 | 0.916 |
| 31 | Isthmus cingulate | -0.243 | -2.029 -- 1.544 | 0.899 |
| 32 | Paracentral | -0.24 | -2.626 -- 2.145 | 1.2 |
| 33 | Caudal anterior cingulate | -0.096 | -2.604 -- 2.412 | 1.262 |

**Footnote:**  Predicted cortical thickness reduction and subcortical volume loss at 12 months in MRI-CBD. Values were derived from linear mixed-effects models

**Abbreviations:** CBD, corticobasal degeneration; CI, confidence interval; ROI, region of interest; SE, standard error.

**Supplementary Table 10. Predicted atrophy at 12 months – RS**

|  | **ROI** | **Predicted atrophy** | **95% CI** | **SE** |
| --- | --- | --- | --- | --- |
| 1 | Superior cerebellar peduncle | -9.335 | -13.704 -- -4.965 | 2.203 |
| 2 | Midbrain | -5.904 | -6.867 -- -4.941 | 0.485 |
| 3 | Entorhinal | -5.006 | -7.45 -- -2.562 | 1.232 |
| 4 | Medulla | -3.549 | -5.744 -- -1.354 | 1.107 |
| 5 | Pons | -2.928 | -3.627 -- -2.228 | 0.353 |
| 6 | Superior temporal | -2.393 | -3.472 -- -1.314 | 0.544 |
| 7 | Transverse temporal | -2.333 | -4.216 -- -0.45 | 0.949 |
| 8 | Fusiform | -1.719 | -2.97 -- -0.467 | 0.631 |
| 9 | Middle temporal | -1.699 | -2.682 -- -0.716 | 0.496 |
| 10 | Pars triangularis | -1.573 | -2.696 -- -0.449 | 0.566 |
| 11 | Insula | -1.319 | -2.817 -- 0.178 | 0.755 |
| 12 | Inferior temporal | -1.31 | -2.351 -- -0.269 | 0.525 |
| 13 | Superior frontal | -1.204 | -2.288 -- -0.12 | 0.547 |
| 14 | Lateral occipital | -1.195 | -2.127 -- -0.263 | 0.47 |
| 15 | Precentral | -1.16 | -3.216 -- 0.897 | 1.037 |
| 16 | Caudal middle frontal | -1.127 | -2.468 -- 0.215 | 0.676 |
| 17 | Posterior cingulate | -1.065 | -2.548 -- 0.418 | 0.748 |
| 18 | Pars opercularis | -1.061 | -2.275 -- 0.154 | 0.612 |
| 19 | Parahippocampal | -0.875 | -2.58 -- 0.83 | 0.86 |
| 20 | Rostral middle frontal | -0.875 | -2.32 -- 0.57 | 0.729 |
| 21 | Rostral anterior cingulate | -0.869 | -2.908 -- 1.171 | 1.028 |
| 22 | Supramarginal | -0.815 | -2.151 -- 0.521 | 0.674 |
| 23 | Banks of the superior temporal sulcus | -0.759 | -2.036 -- 0.519 | 0.644 |
| 24 | Paracentral | -0.664 | -2.657 -- 1.328 | 1.005 |
| 25 | Pars orbitalis | -0.416 | -1.767 -- 0.935 | 0.681 |
| 26 | Postcentral | -0.41 | -1.818 -- 0.999 | 0.71 |
| 27 | Frontal pole | -0.397 | -3.252 -- 2.458 | 1.44 |
| 28 | Temporal pole | -0.252 | -2.984 -- 2.481 | 1.378 |
| 29 | Inferior parietal | -0.162 | -1.258 -- 0.934 | 0.552 |
| 30 | Precuneus | -0.066 | -1.552 -- 1.421 | 0.749 |

**Footnote:**  Predicted cortical thickness reduction and subcortical volume loss at 12 months in RS. Values were derived from linear mixed-effects models

**Abbreviations:** CI, confidence interval; ROI, region of interest; RS, Richardson’s syndrome; SE, standard error.

**Supplementary Table 11. Predicted atrophy at 12 months – CBS**

|  | **ROI** | **Predicted atrophy** | **95% CI** | **SE** |
| --- | --- | --- | --- | --- |
| 1 | Midbrain | -4.497 | -6.2 -- -2.793 | 0.86 |
| 2 | Precentral | -3.359 | -5.851 -- -0.866 | 1.258 |
| 3 | Superior cerebellar peduncle | -3.311 | -7.57 -- 0.948 | 2.149 |
| 4 | Supramarginal | -2.634 | -3.678 -- -1.59 | 0.527 |
| 5 | Caudal middle frontal | -2.562 | -3.889 -- -1.236 | 0.669 |
| 6 | Frontal pole | -2.355 | -4.575 -- -0.134 | 1.12 |
| 7 | Entorhinal | -2.327 | -4.259 -- -0.395 | 0.975 |
| 8 | Superior frontal | -2.28 | -3.314 -- -1.246 | 0.522 |
| 9 | Transverse temporal | -2.232 | -4.224 -- -0.24 | 1.005 |
| 10 | Parahippocampal | -2.14 | -3.868 -- -0.411 | 0.872 |
| 11 | Paracentral | -2.06 | -4.186 -- 0.067 | 1.073 |
| 12 | Rostral middle frontal | -1.972 | -3.346 -- -0.598 | 0.693 |
| 13 | Pars triangularis | -1.826 | -3.266 -- -0.387 | 0.726 |
| 14 | Pars opercularis | -1.694 | -3.011 -- -0.378 | 0.664 |
| 15 | Insula | -1.597 | -3.024 -- -0.171 | 0.72 |
| 16 | Temporal pole | -1.536 | -3.616 -- 0.544 | 1.049 |
| 17 | Fusiform | -1.531 | -2.949 -- -0.114 | 0.715 |
| 18 | Middle temporal | -1.525 | -2.358 -- -0.692 | 0.421 |
| 19 | Superior temporal | -1.461 | -2.42 -- -0.502 | 0.484 |
| 20 | Superior parietal | -1.366 | -2.735 -- 0.002 | 0.691 |
| 21 | Banks of the superior temporal sulcus | -1.303 | -2.636 -- 0.029 | 0.672 |
| 22 | Caudal anterior cingulate | -1.181 | -3.179 -- 0.817 | 1.008 |
| 23 | Precuneus | -1.162 | -2.438 -- 0.114 | 0.644 |
| 24 | Posterior cingulate | -1.074 | -2.427 -- 0.28 | 0.683 |
| 25 | Inferior temporal | -1.055 | -2.024 -- -0.085 | 0.489 |
| 26 | Inferior parietal | -0.961 | -2.062 -- 0.14 | 0.556 |
| 27 | Pons | -0.934 | -1.8 -- -0.068 | 0.437 |
| 28 | Postcentral | -0.658 | -2.292 -- 0.976 | 0.825 |
| 29 | Isthmus cingulate | -0.639 | -2.036 -- 0.757 | 0.705 |
| 30 | Lateral occipital | -0.627 | -1.833 -- 0.579 | 0.609 |
| 31 | Lateral orbitofrontal | -0.617 | -2.402 -- 1.168 | 0.901 |
| 32 | Pars orbitalis | -0.432 | -1.913 -- 1.049 | 0.747 |
| 33 | Medial orbitofrontal | -0.34 | -1.818 -- 1.138 | 0.746 |
| 34 | Cuneus | -0.304 | -1.67 -- 1.061 | 0.689 |
| 35 | Pericalcarine | -0.07 | -2.716 -- 2.577 | 1.335 |

**Footnote:**  Predicted cortical thickness reduction and subcortical volume loss at 12 months in CBS. Values were derived from linear mixed-effects models

**Abbreviations:** CBS, corticobasal syndrome; CI, confidence interval; ROI, region of interest; SE, standard error.

**Supplementary Table 12. Data-driven top ten MRI-signature in MRI-PSP**

|  | **Combination** | **Estimated sample size** |
| --- | --- | --- |
| 1 | Midbrain + Superior temporal + Pons + Rostral middle frontal | 152 |
| 2 | Midbrain + Superior temporal + Rostral middle frontal | 158 |
| 3 | Midbrain + Superior temporal + Pons + Posterior cingulate + Rostral middle frontal | 161 |
| 4 | Midbrain + Superior temporal + Pons + Rostral middle frontal + Lateral occipital | 162 |
| 5 | Midbrain + Pars opercularis | 164 |
| 6 | Midbrain + Superior temporal + Pons + Pars opercularis + Rostral middle frontal | 164 |
| 7 | Midbrain + Superior temporal + Pars opercularis | 167 |
| 8 | Midbrain + Pons + Pars opercularis | 168 |
| 9 | Midbrain + Superior temporal + Pons + Pars opercularis | 170 |
| 10 | Midbrain + Superior temporal | 172 |

**Footnote:** The most efficient data-driven combinations of ROIs to detect 30% reduction in atrophy at 12 months with 10% attrition rate were derived from linear mixed-effects models.

**Abbreviations:** MRI, magnetic resonance imaging; PSP, progressive supranuclear palsy.

**Supplementary Table 13. Data-driven top ten MRI-signature in MRI-CBD**

|  | **Combination** | **Estimated sample size** |
| --- | --- | --- |
| 1 | Midbrain + Pons  + Superior frontal + Insula + Bank of the superior temporal sulcus | 81 |
| 2 | Midbrain + Pons  + Superior frontal + Insula | 82 |
| 3 | Midbrain + Pons + Superior frontal + Insula + Middle temporal | 87 |
| 4 | Midbrain + Pons  + Superior frontal + Entorhinal | 87 |
| 5 | Midbrain + Pons  + Superior frontal + Insula + Lateral occipital | 88 |
| 6 | Midbrain + Pons  + Insula + Bank of the superior temporal sulcus | 91 |
| 7 | Midbrain + Pons  + Superior frontal + Insula + Inferior temporal | 91 |
| 8 | Midbrain + Pons  + Superior frontal + Insula + Superior temporal | 94 |
| 9 | Midbrain  + Superior frontal + Insula | 96 |
| 10 | Midbrain + Pons  + Middle temporal + Entorhinal | 99 |

**Footnote:** The most efficient data-driven combinations of ROIs to detect 30% reduction in atrophy at 12 months with 10% attrition rate were derived from linear mixed-effects models.

**Abbreviations:** CBD, corticobasal degeneration; MRI, magnetic resonance imaging.

**Supplementary Table 14. Data-driven top ten MRI-signature in RS**

|  | **Combination** | **Estimated sample size** |
| --- | --- | --- |
| 1 | Midbrain + Pons + Pars opercularis | 89 |
| 2 | Midbrain + Pons + Pars orbitalis | 91 |
| 3 | Midbrain + Pons + Pars triangularis | 92 |
| 4 | Midbrain + Pons + Superior temporal + Pars orbitalis | 104 |
| 5 | Midbrain + Pons + Middle temporal + Pars orbitalis | 106 |
| 6 | Midbrain + Pons + Caudal middle frontal + Pars orbitalis | 107 |
| 7 | Midbrain + Pons + Superior temporal + Rostral middle frontal | 109 |
| 8 | Midbrain + Pons + Pars opercularis + Lateral occipital | 109 |
| 9 | Midbrain + Pons | 109 |
| 10 | Midbrain + Pons + Pars orbitalis + Pars opercularis | 110 |

**Footnote:** The most efficient data-driven combinations of ROIs to detect 30% reduction in atrophy at 12 months with 10% attrition rate were derived from linear mixed-effects models.

**Abbreviations:** MRI, magnetic resonance imaging; RS, Richardson’s syndrome.

**Supplementary Table 15. Data-driven top ten MRI-signature in CBS**

|  | **Combination** | **Estimated sample size** |
| --- | --- | --- |
| 1 | Midbrain + Supramarginal + Entorhinal + Superior frontal | 164 |
| 2 | Midbrain + Caudal middle frontal + Entorhinal + Superior frontal + Isthmus cingulate | 164 |
| 3 | Midbrain + Supramarginal + Entorhinal + Superior frontal + Isthmus cingulate | 170 |
| 4 | Midbrain + Supramarginal + Entorhinal + Superior frontal + Pons | 176 |
| 5 | Midbrain + Entorhinal + Superior frontal | 182 |
| 6 | Midbrain + Supramarginal + Entorhinal + Superior frontal + Middle temporal | 183 |
| 7 | Midbrain + Supramarginal + Entorhinal + Superior frontal + Insula | 184 |
| 8 | Midbrain + Entorhinal + Superior frontal + Isthmus cingulate | 190 |
| 9 | Midbrain + Entorhinal + Superior frontal + Middle temporal | 191 |
| 10 | Midbrain + Entorhinal + Superior frontal + Pons + Isthmus cingulate | 194 |

**Footnote:** The most efficient data-driven combinations of ROIs to detect 30% reduction in atrophy at 12 months with 10% attrition rate were derived from linear mixed-effects models.

**Abbreviations:** CBS, corticobasal syndrome; MRI, magnetic resonance imaging.

**Supplementary Table 16. Linear mixed effect model for midbrain volume after ComBat harmonization**

| **Before enriched selection** | | | | | | **After enriched selection** | | | | | |
| --- | --- | --- | --- | --- | --- | --- | --- | --- | --- | --- | --- |
|  | **Estimate** | **Std. Error** | **df** | **t value** | **p-value** |  | **Estimate** | **Std. Error** | **df** | **t value** | **p-value** |
| **Intercept** | 0.775 | 0.869 | 170.325 | 0.891 | 0.374 | **Intercept** | 0.037 | 1.057 | 178.108 | 0.035 | 0.972 |
| **Time (months)** | -0.486 | 0.061 | 125.201 | -7.935 | **<0.001** | **Time (months)** | -0.443 | 0.056 | 126.092 | -7.954 | **<0.001** |
| **Sex (male)** | -0.058 | 0.263 | 173.026 | -0.223 | 0.824 | **Sex (male** | -0.131 | 0.265 | 177.86 | -0.493 | 0.623 |
| **Basal midbrain volume** | 0 | 0 | 172.713 | -0.919 | 0.36 | **Basal midbrain volume** | 0 | 0 | 180.802 | 0.022 | 0.983 |
| **Group (CBS)** | -0.045 | 0.264 | 137.037 | -0.169 | 0.866 | **Group (MRI-CBD)** | -0.236 | 0.347 | 151.331 | -0.682 | 0.497 |
| **Group (nfvPPA)** | 0.473 | 0.524 | 134.115 | 0.902 | 0.369 | **Group (AD)** | -0.279 | 0.44 | 140.661 | -0.635 | 0.526 |
| **Time:Group (CBS)** | 0.123 | 0.086 | 125.317 | 1.433 | 0.154 | **Time:Group (MRI-CBD)** | -0.097 | 0.085 | 126.996 | -1.144 | 0.255 |
| **Time:Group (nfvPPA)** | 0.001 | 0.176 | 167.88 | 0.003 | 0.997 | **Time:Group (AD)** | 0.378 | 0.12 | 124.964 | 3.149 | **0.002** |

**Footnote:**  Linear mixed-effects models showing the longitudinal change in midbrain volume according to clinical diagnosis only and after the enriched selection using AD biomarkers and multinomial logistic regression model.

**Abbreviations:** AD, Alzheimer’s disease; CBD, corticobasal degeneration; CBS, corticobasal syndrome; df, degrees of freedom; MRI, magnetic resonance imaging; nfvPPA, non-fluent variant of primary progressive aphasia.

**Supplementary Table 17. Sample size estimation after ComBat harmonization**

|  | **RS** | **MRI-PSP** | **CBS** | **MRI-CBD** |
| --- | --- | --- | --- | --- |
| **Midbrain (% change)** | 100 (92-106) | 236 (223-245) | 659 (604-692) | 112 (103-120) |
| **MRI signature** | 103 (98-111) | 151 (143-159) | 326 (307-339) | 81 (76-85) |

The table shows the bootstrapped estimated sample size and 95% confidence interval after jackknife bootstrapping for a clinical trial (30% reduction at 12 months with 10% attrition).

**Abbreviations:** CBD, corticobasal degeneration; CBS, corticobasal syndrome; MRI, magnetic resonance imaging; PSP, progressive supranuclear palsy; RS, Richardson’s syndrome.

**Supplementary Table 18. Subsample of participants in 4RTNI excluding participants with more than 5 years of symptoms**

| **Characteristic** | **All, N = 75** | **MRI-PSP, N = 37** | **MRI-CBD, N = 26** | **AD, N = 12** | **Adjusted p-value** |
| --- | --- | --- | --- | --- | --- |
| **Age** | 67 (9) | 69 (7) | 64 (10) | 67 (10) | >0.9 |
| **Biological Sex** |  | | | | >0.9 |
| Woman | 39 (52%) | 22 (59%) | 12 (46%) | 5 (42%) |  |
| Man | 36 (48%) | 15 (41%) | 14 (54%) | 7 (58%) |  |
| **Number of visits** |  | | | | >0.9 |
| 2 | 16 (21%) | 10 (27%) | 4 (15%) | 2 (17%) |  |
| 3 | 47 (63%) | 21 (57%) | 19 (73%) | 7 (58%) |  |
| >3 | 12 (16%) | 6 (16%) | 3 (12%) | 3 (25%) |  |
| **Clinical phenotype** |  | | | | **<0.001** |
| RS | 31 (41%) | 29 (78%) | 2 (7.7%) | 0 (0%) |  |
| CBS | 38 (51%) | 8 (22%) | 20 (77%) | 10 (83%) |  |
| nfvPPA | 6 (8.0%) | 0 (0%) | 4 (15%) | 2 (17%) |  |
| **PSPRS** | 24 (14) | 31 (14) | 20 (10) | 15 (11) | **0.004** |
| **SEADL** | 64 (23) | 61 (23) | 67 (23) | 68 (21) | >0.9 |
| **Midbrain volume** | 5,382 (803) | 4,846 (540) | 5,847 (691) | 6,032 (600) | **<0.001** |
| **Stability of MRI-based classification** |  | | | | **0.004** |
| CBD to PSP | 8 (11%) | 0 (0%) | 8 (31%) | 0 (0%) |  |
| Stable | 67 (89%) | 37 (100%) | 18 (69%) | 12 (100%) |  |

**Footnote:** values reported are mean (standard deviation) or n (%). Bonferroni correction for multiple testing was applied.

**Abbreviations:** AD, Alzheimer’s disease; CBD, corticobasal degeneration; CBS, corticobasal syndrome; MRI, magnetic resonance imaging; nfvPPA, non-fluent variant of primary progressive aphasia; PSP, progressive supranuclear palsy; PSPRS, progressive supranuclear palsy rating scale; RS, Richardson’s syndrome; SEADL, Schwab and England Activities of Daily Living scale.

**Supplementary Table 19. Sample size estimation in DAV**

|  | **3T MRI scans** | | **All MRI scans** | |
| --- | --- | --- | --- | --- |
|  | **RS (n = 100)** | **PSP-MRI (n = 85)** | **RS (n = 162)** | **PSP-MRI (n = 138)** |
| **PSPRS** | 147 (141-150) | 150 (143-154) | 141 (138-143) | 143 (139-145) |
| **Midbrain (% change)** | 343 (317-354) | 302 (290-328) | 393 (376-401) | 388 (387-417) |
| **PSP MRI signature** | 171 (164-175) | 146 (140-150) | 181 (178-184) | 166 (163-169) |

**Footnote:** Sample size estimation and 95% confidence interval after jackknife bootstrapping for hypothetical clinical trials to detect 30% reduction in each outcome at 12 months with 10% attrition rate.

**Abbreviations**: DAV, Davunetide trial cohort; MRI, magnetic resonance imaging; PSP, progressive supranuclear palsy; PSPRS, progressive supranuclear palsy rating scale.
