# Supplementary material for: Potential role of MRI to optimize clinical trial design for progressive supranuclear palsy and corticobasal degeneration
Source: J Prev Alzheimers Dis. 2026 Jan 24;13(3):100486. doi: 10.1016/j.tjpad.2026.100486 (PMC12860716; doi:10.1016/j.tjpad.2026.100486)
Supplement: Supplementary file 2 [file mmc2.docx]

**Sample characteristics (after ComBat harmonization)**

|  | **4RTNI** | | | | | **DAV** | | | |
| --- | --- | --- | --- | --- | --- | --- | --- | --- | --- |
| **Characteristic** | **All, N = 106** | **MRI-PSP, N = 51** | **MRI-CBD, N = 41** | **AD, N = 14** | **Adjusted p-value** | **All, N = 100** | **MRI-PSP, N = 85** | **MRI-CBD, N = 15** | **p-value** |
| **Age** | 68  (8) | 70  (8) | 65  (9) | 67  (10) | >0.9 | 67  (7) | 68  (6) | 61  (8) | **0.003** |
| **Biological Sex** |  | | | | >0.9 |  | | | 0.2 |
| Woman | 53 (50%) | 31 (61%) | 17 (41%) | 5 (36%) |  | 54 (54%) | 48 (56%) | 6 (40%) |  |
| Man | 53 (50%) | 20 (39%) | 24 (59%) | 9 (64%) |  | 46 (46%) | 37 (44%) | 9 (60%) |  |
| **Number of visits** |  | | | | >0.9 |  | | | |
| 2 | 23 (22%) | 16 (31%) | 5 (12%) | 2 (14%) |  | 100 (100%) | 85 (100%) | 15 (100%) |  |
| 3 | 63 (59%) | 25 (49%) | 29 (71%) | 9 (64%) |  |  | | |  |
| >3 | 20 (19%) | 10 (20%) | 7 (17%) | 3 (21%) |  |  |  |  |  |
| **Clinical phenotype** |  | | | | **<0.001** |  | | | |
| RS | 47 (44%) | 39 (76%) | 8 (20%) | 0  (0%) |  | 100 (100%) | 85 (100%) | 15 (100%) |  |
| CBS | 51 (48%) | 12 (24%) | 27 (66%) | 12 (86%) |  |  | | |  |
| nfvPPA | 8  (8%) | 0  (0%) | 6 (14%) | 2 (14%) |  |  |  |  |  |
| **Years of symptoms** |  | | | | >0.9 |  | | | >0.9 |
| <= 5 Years | 75 (72%) | 39 (72%) | 36 (72%) | 75 (72%) |  | 86 (95%) | 72 (94%) | 14 (100%) |  |
| > 5 Years | 29 (28%) | 15 (28%) | 14 (28%) | 29 (28%) |  | 5  (5%) | 5  (6%) | 0  (0%) |  |
| **PSPRS** | 26 (15) | 33  (14) | 21 (13) | 15  (11) | **<0.001** | 38  (12) | 40  (11) | 29  (18) | **0.003** |
| **SEADL** | 63 (24) | 59  (25) | 66 (22) | 70  (21) | >0.9 | 54  (25) | 51  (24) | 71  (26) | **0.005** |
| **Midbrain volume** | 5,408 (803) | 4,827 (545) | 5,946 (615) | 5,951 (595) | **<0.001** | 4,836 (683) | 4,695 (553) | 5,630 (819) | **<0.001** |
| **Stability of MRI-based classification** |  | | | | **<0.001** |  | | | **<0.001** |
| CBD to PSP | 14 (13%) | 0  (0%) | 14 (34%) | 0  (0%) |  | 4  (4%) | 0  (0%) | 4 (27%) |  |
| PSP to CBD |  | | | |  | 1  (1%) | 1  (1%) | 0  (0%) |  |
| Stable | 92 (87%) | 51 (100%) | 27 (66%) | 14 (100%) |  | 95 (95%) | 84 (99%) | 11 (73%) |  |

**Footnote:** values reported are mean (standard deviation) or n (%). Bonferroni correction for multiple testing was applied in 4RTNI.

**Abbreviations:** AD, Alzheimer’s disease; CBD, corticobasal degeneration; CBS, corticobasal syndrome; MRI, magnetic resonance imaging; nfvPPA, non-fluent variant of primary progressive aphasia; PSP, progressive supranuclear palsy; PSPRS, progressive supranuclear palsy rating scale; RS, Richardson’s syndrome; SEADL, Schwab and England Activities of Daily Living scale.

**Predicted atrophy at 12 months - MRI-PSP (after ComBat harmonization)**

|  | **ROI** | **Predicted atrophy** | **95% CI** | **SE** |
| --- | --- | --- | --- | --- |
| 1 | Superior cerebellar peduncle | -7.946 | -12.753 -- -3.138 | 2.426 |
| 2 | Midbrain | -5.230 | -6.423 -- -4.038 | 0.602 |
| 3 | Entorhinal | -4.962 | -7.382 -- -2.542 | 1.221 |
| 4 | Transverse temporal | -2.516 | -4.371 -- -0.660 | 0.936 |
| 5 | Pons | -2.411 | -3.204 -- -1.618 | 0.400 |
| 6 | Superior temporal | -2.384 | -3.390 -- -1.378 | 0.508 |
| 7 | Medulla | -2.280 | -5.192 -- 0.633 | 1.470 |
| 8 | Pars triangularis | -2.173 | -3.478 -- -0.868 | 0.659 |
| 9 | Paracentral | -1.951 | -3.943 -- 0.040 | 1.005 |
| 10 | Precentral | -1.930 | -3.533 -- -0.328 | 0.809 |
| 11 | Fusiform | -1.908 | -3.194 -- -0.621 | 0.649 |
| 12 | Middle temporal | -1.866 | -2.810 -- -0.922 | 0.477 |
| 13 | Caudal middle frontal | -1.833 | -2.935 -- -0.731 | 0.556 |
| 14 | Frontal pole | -1.712 | -4.628 -- 1.205 | 1.472 |
| 15 | Pars opercularis | -1.544 | -2.512 -- -0.576 | 0.488 |
| 16 | Rostral middle frontal | -1.510 | -2.930 -- -0.089 | 0.717 |
| 17 | Posterior cingulate | -1.440 | -2.883 -- -0.003 | 0.728 |
| 18 | Superior frontal | -1.420 | -2.487 -- -0.353 | 0.538 |
| 19 | Insula | -1.390 | -2.817 -- 0.038 | 0.720 |
| 20 | Inferior temporal | -1.369 | -2.445 -- -0.293 | 0.543 |
| 21 | Supramarginal | -1.361 | -2.531 -- -0.191 | 0.590 |
| 22 | Temporal pole | -1.360 | -3.812 – 1.092 | 1.237 |
| 23 | Lateral occipital | -1.253 | -2.360 -- -0.145 | 0.559 |
| 24 | Pars orbitalis | -1.030 | -2.403 -- 0.342 | 0.693 |
| 25 | Parahippocampal | -0.883 | -2.576 -- 0.811 | 0.855 |
| 26 | Banks of the superior temporal sulcus | -0.859 | -2.046 -- 0.327 | 0.599 |
| 27 | Caudal anterior cingulate | -0.835 | -2.799 -- 1.130 | 0.991 |
| 28 | Rostral anterior cingulate | -0.723 | -2.686 -- 1.239 | 0.990 |
| 29 | Postcentral | -0.170 | -1.499 -- 1.159 | 0.671 |
| 30 | Lingual | -0.143 | -1.369 -- 1.082 | 0.618 |

**Footnote:**  Predicted cortical thickness reduction and subcortical volume loss at 12 months in MRI-PSP. Values were derived from linear mixed-effects models

**Abbreviations:** CI, confidence interval; PSP, progressive supranuclear palsy; ROI, region of interest; SE, standard error.

**Predicted atrophy at 12 months - MRI-CBD (after ComBat harmonization)**

|  | **ROI** | **Predicted atrophy** | **95% CI** | **SE** |
| --- | --- | --- | --- | --- |
| 1 | Midbrain | -6.524 | -7.818 -- -5.229 | 0.651 |
| 2 | Superior cerebellar peduncle | -3.564 | -7.618 -- 0.490 | 2.040 |
| 3 | Parahippocampal | -2.360 | -4.124 -- -0.595 | 0.888 |
| 4 | Pons | -2.245 | -3.071 -- -1.419 | 0.416 |
| 5 | Supramarginal | -2.174 | -3.854 -- -0.494 | 0.845 |
| 6 | Superior frontal | -2.118 | -3.365 -- -0.870 | 0.628 |
| 7 | Precentral | -1.950 | -5.588 -- 1.688 | 1.830 |
| 8 | Insula | -1.929 | -3.742 -- -0.117 | 0.912 |
| 9 | Frontal pole | -1.855 | -4.148 -- 0.438 | 1.154 |
| 10 | Caudal middle frontal | -1.636 | -3.884 -- 0.611 | 1.131 |
| 11 | Banks of the superior temporal sulcus | -1.629 | -2.980 -- -0.278 | 0.680 |
| 12 | Rostral middle frontal | -1.616 | -3.407 – 0.176 | 0.901 |
| 13 | Postcentral | -1.615 | -4.137 -- 0.906 | 1.268 |
| 14 | Pars opercularis | -1.595 | -3.361 -- 0.171 | 0.888 |
| 15 | Pars triangularis | -1.553 | -3.197 -- 0.091 | 0.827 |
| 16 | Inferior parietal | -1.486 | -2.724 -- -0.247 | 0.623 |
| 17 | Transverse temporal | -1.424 | -3.365 -- 0.516 | 0.976 |
| 18 | Middle temporal | -1.362 | -2.189 -- -0.534 | 0.416 |
| 19 | Precuneus | -1.272 | -2.768 -- 0.224 | 0.753 |
| 20 | Entorhinal | -1.202 | -3.328 -- 0.924 | 1.070 |
| 21 | Superior temporal | -1.118 | -2.132 -- -0.104 | 0.510 |
| 22 | Superior parietal | -1.029 | -2.636 -- 0.578 | 0.808 |
| 23 | Posterior cingulate | -0.893 | -2.413 -- 0.627 | 0.765 |
| 24 | Inferior temporal | -0.887 | -1.810 -- 0.037 | 0.465 |
| 25 | Cerebellum cortex | -0.812 | -3.204 -- 1.581 | 1.204 |
| 26 | Fusiform | -0.696 | -2.135 -- 0.742 | 0.724 |
| 27 | Lateral occipital | -0.663 | -1.728 -- 0.401 | 0.536 |
| 28 | Cuneus | -0.634 | -2.199 -- 0.930 | 0.787 |
| 29 | Pars orbitalis | -0.493 | -2.371 -- 1.385 | 0.945 |
| 30 | Lateral orbitofrontal | -0.449 | -2.260 -- 1.363 | 0.911 |
| 31 | Paracentral | -0.254 | -2.625 -- 2.117 | 1.193 |
| 32 | Isthmus cingulate | -0.236 | -2.014 – 1.543 | 0.895 |
| 33 | Caudal anterior cingulate | -0.093 | -2.590 -- 2.404 | 1.256 |

**Footnote:**  Predicted cortical thickness reduction and subcortical volume loss at 12 months in MRI-CBD. Values were derived from linear mixed-effects models

**Abbreviations:** CBD, corticobasal degeneration; CI, confidence interval; ROI, region of interest; SE, standard error.

**Predicted atrophy at 12 months – RS (after ComBat harmonization)**

|  | **ROI** | **Predicted atrophy** | **95% CI** | **SE** |
| --- | --- | --- | --- | --- |
| 1 | Superior cerebellar peduncle | -9.338 | -13.701 -- -4.975 | 2.200 |
| 2 | Midbrain | -5.907 | -6.880 -- -4.935 | 0.490 |
| 3 | Entorhinal | -5.007 | -7.447 -- -2.567 | 1.230 |
| 4 | Medulla | -3.574 | -5.767 -- -1.380 | 1.106 |
| 5 | Pons | -2.937 | -3.634 -- -2.240 | 0.351 |
| 6 | Superior temporal | -2.387 | -3.460 -- -1.313 | 0.541 |
| 7 | Transverse temporal | -2.338 | -4.223 -- -0.452 | 0.951 |
| 8 | Fusiform | -1.718 | -2.967 -- -0.468 | 0.630 |
| 9 | Middle temporal | -1.698 | -2.682 -- -0.714 | 0.496 |
| 10 | Pars triangularis | -1.569 | -2.694 -- -0.444 | 0.567 |
| 11 | Inferior temporal | -1.311 | -2.354 -- -0.268 | 0.526 |
| 12 | Insula | -1.308 | -2.800 – 0.183 | 0.752 |
| 13 | Superior frontal | -1.200 | -2.286 -- -0.115 | 0.547 |
| 14 | Lateral occipital | -1.193 | -2.122 -- -0.263 | 0.468 |
| 15 | Precentral | -1.157 | -3.211 -- 0.897 | 1.036 |
| 16 | Caudal middle frontal | -1.125 | -2.466 -- 0.217 | 0.676 |
| 17 | Posterior cingulate | -1.063 | -2.547 -- 0.421 | 0.748 |
| 18 | Pars opercularis | -1.059 | -2.271 -- 0.153 | 0.611 |
| 19 | Parahippocampal | -0.887 | -2.594 -- 0.819 | 0.860 |
| 20 | Rostral middle frontal | -0.876 | -2.322 -- 0.570 | 0.729 |
| 21 | Rostral anterior cingulate | -0.859 | -2.898 -- 1.180 | 1.028 |
| 22 | Supramarginal | -0.809 | -2.140 -- 0.522 | 0.671 |
| 23 | Banks of the superior temporal sulcus | -0.755 | -2.029 -- 0.520 | 0.643 |
| 24 | Paracentral | -0.658 | -2.644 -- 1.328 | 1.001 |
| 25 | Pars orbitalis | -0.414 | -1.766 -- 0.938 | 0.682 |
| 26 | Postcentral | -0.403 | -1.811 – 1.005 | 0.710 |
| 27 | Frontal pole | -0.379 | -3.222 -- 2.463 | 1.433 |
| 28 | Temporal pole | -0.244 | -2.974 -- 2.486 | 1.376 |
| 29 | Inferior parietal | -0.167 | -1.261 -- 0.927 | 0.552 |
| 30 | Precuneus | -0.056 | -1.536 -- 1.423 | 0.746 |

**Footnote:**  Predicted cortical thickness reduction and subcortical volume loss at 12 months in RS. Values were derived from linear mixed-effects models

**Abbreviations:** CI, confidence interval; ROI, region of interest; RS, Richardson’s syndrome; SE, standard error.

**Predicted atrophy at 12 months – CBS (after ComBat harmonization)**

|  | **ROI** | **Predicted atrophy** | **95% CI** | **SE** |
| --- | --- | --- | --- | --- |
| 1 | Midbrain | -4.508 | -6.198 -- -2.817 | 0.853 |
| 2 | Precentral | -3.370 | -5.851 -- -0.887 | 1.253 |
| 3 | Superior cerebellar peduncle | -3.298 | -7.557 -- 0.961 | 2.149 |
| 4 | Supramarginal | -2.629 | -3.672 -- -1.585 | 0.526 |
| 5 | Caudal middle frontal | -2.559 | -3.881 -- -1.238 | 0.667 |
| 6 | Frontal pole | -2.362 | -4.576 -- -0.147 | 1.117 |
| 7 | Entorhinal | -2.314 | -4.239 -- -0.390 | 0.971 |
| 8 | Superior frontal | -2.276 | -3.307 -- -1.245 | 0.520 |
| 9 | Transverse temporal | -2.209 | -4.193 -- -0.225 | 1.001 |
| 10 | Parahippocampal | -2.127 | -3.844 -- -0.411 | 0.866 |
| 11 | Paracentral | -2.062 | -4.177 -- 0.052 | 1.067 |
| 12 | Rostral middle frontal | -1.958 | -3.324 -- -0.591 | 0.689 |
| 13 | Pars triangularis | -1.818 | -3.253 -- -0.384 | 0.724 |
| 14 | Pars opercularis | -1.693 | -3.008 -- -0.379 | 0.663 |
| 15 | Insula | -1.589 | -3.005 -- -0.174 | 0.714 |
| 16 | Fusiform | -1.521 | -2.930 -- -0.111 | 0.711 |
| 17 | Middle temporal | -1.521 | -2.351 -- -0.691 | 0.419 |
| 18 | Temporal pole | -1.516 | -3.585 – 0.554 | 1.044 |
| 19 | Superior temporal | -1.456 | -2.41 -- -0.503 | 0.481 |
| 20 | Superior parietal | -1.362 | -2.726 -- 0.001 | 0.688 |
| 21 | Banks of the superior temporal sulcus | -1.296 | -2.619 -- 0.026 | 0.667 |
| 22 | Caudal anterior cingulate | -1.175 | -3.163 -- 0.813 | 1.003 |
| 23 | Precuneus | -1.160 | -2.433 -- 0.114 | 0.643 |
| 24 | Posterior cingulate | -1.066 | -2.417 -- 0.285 | 0.682 |
| 25 | Inferior temporal | -1.048 | -2.012 -- -0.085 | 0.486 |
| 26 | Inferior parietal | -0.957 | -2.055 -- 0.141 | 0.554 |
| 27 | Pons | -0.947 | -1.808 -- -0.085 | 0.435 |
| 28 | Postcentral | -0.664 | -2.296 -- 0.968 | 0.824 |
| 29 | Isthmus cingulate | -0.634 | -2.026 -- 0.757 | 0.702 |
| 30 | Lateral occipital | -0.622 | -1.821 -- 0.578 | 0.605 |
| 31 | Lateral orbitofrontal | -0.603 | -2.381 -- 1.175 | 0.897 |
| 32 | Pars orbitalis | -0.422 | -1.895 -- 1.052 | 0.744 |
| 33 | Medial orbitofrontal | -0.331 | -1.800 -- 1.138 | 0.741 |
| 34 | Cuneus | -0.301 | -1.664 -- 1.062 | 0.688 |
| 35 | Pericalcarine | -0.065 | -2.702 -- 2.572 | 1.331 |

**Footnote:**  Predicted cortical thickness reduction and subcortical volume loss at 12 months in CBS. Values were derived from linear mixed-effects models

**Abbreviations:** CBS, corticobasal syndrome; CI, confidence interval; ROI, region of interest; SE, standard error.

**Data-driven top ten MRI-signature in MRI-PSP (after ComBat harmonization)**

|  | **Combination** | **Estimated sample size** |
| --- | --- | --- |
| 1 | Midbrain + Superior temporal + Pons + Rostral middle frontal | 151 |
| 2 | Midbrain + Superior temporal + Rostral middle frontal | 158 |
| 3 | Midbrain + Superior temporal + Pons + Posterior cingulate + Rostral middle frontal | 159 |
| 4 | Midbrain + Superior temporal + Pons + Rostral middle frontal + Lateral occipital | 161 |
| 5 | Midbrain + Pars opercularis | 163 |
| 6 | Midbrain + Superior temporal + Pons + Pars opercularis + Rostral middle frontal | 163 |
| 7 | Midbrain + Superior temporal + Pars opercularis | 166 |
| 8 | Midbrain + Pons + Pars opercularis | 168 |
| 9 | Midbrain + Superior temporal + Pons + Pars opercularis | 169 |
| 10 | Midbrain + Superior temporal | 170 |

**Footnote:** The most efficient data-driven combinations of ROIs to detect 30% reduction in atrophy at 12 months with 10% attrition rate were derived from linear mixed-effects models.

**Abbreviations:** MRI, magnetic resonance imaging; PSP, progressive supranuclear palsy.

**Data-driven top ten MRI-signature in MRI-CBD (after ComBat harmonization)**

|  | **Combination** | **Estimated sample size** |
| --- | --- | --- |
| 1 | Midbrain + Pons  + Superior frontal + Insula + Bank of the superior temporal sulcus | 81 |
| 2 | Midbrain + Pons  + Superior frontal + Insula | 82 |
| 3 | Midbrain + Pons  + Superior frontal + Entorhinal | 86 |
| 4 | Midbrain + Pons  + Superior frontal + Insula + Middle temporal | 87 |
| 5 | Midbrain + Pons  + Superior frontal + Insula + Lateral occipital | 88 |
| 6 | Midbrain + Pons  + Superior frontal + Insula + Inferior temporal | 91 |
| 7 | Midbrain + Pons  + Insula + Inferior temporal + Bank of the superior temporal sulcus | 91 |
| 8 | Midbrain + Pons  + Superior frontal + Insula + Superior temporal | 95 |
| 9 | Midbrain  + Superior frontal + Insula | 96 |
| 10 | Midbrain + Pons  + Superior frontal + Middle temporal + Entorhinal | 98 |

**Footnote:** The most efficient data-driven combinations of ROIs to detect 30% reduction in atrophy at 12 months with 10% attrition rate were derived from linear mixed-effects models.

**Abbreviations:** CBD, corticobasal degeneration; MRI, magnetic resonance imaging.

**Data-driven top ten MRI-signature in RS (after ComBat harmonization)**

|  | **Combination** | **Estimated sample size** |
| --- | --- | --- |
| 1 | Midbrain + Pons + Pars opercularis | 83 |
| 2 | Midbrain + Pons + Pars triangularis | 93 |
| 3 | Midbrain + Pons + Middle temporal + Pars opercularis | 93 |
| 4 | Midbrain + Pons + Middle temporal | 97 |
| 5 | Midbrain + Pons | 98 |
| 6 | Midbrain + Pons + Superior frontal | 100 |
| 7 | Midbrain | 100 |
| 8 | Midbrain + Pons + Superior frontal + Posterior cingulate | 102 |
| 9 | Midbrain + Pons + Middle temporal + Superior frontal | 102 |
| 10 | Midbrain + Pons + Superior temporal + Rostral middle frontal | 103 |

**Footnote:** The most efficient data-driven combinations of ROIs to detect 30% reduction in atrophy at 12 months with 10% attrition rate were derived from linear mixed-effects models.

**Abbreviations:** MRI, magnetic resonance imaging; RS, Richardson’s syndrome.

**Data-driven top ten MRI-signature in CBS (after ComBat harmonization)**

|  | **Combination** | **Estimated sample size** |
| --- | --- | --- |
| 1 | Midbrain + Supramarginal + Entorhinal + Superior frontal | 163 |
| 2 | Midbrain + Caudal middle frontal + Entorhinal + Superior frontal + Isthmus cingulate | 163 |
| 3 | Midbrain + Supramarginal + Entorhinal + Superior frontal + Isthmus cingulate | 169 |
| 4 | Midbrain + Supramarginal + Entorhinal + Superior frontal + Pons | 175 |
| 5 | Midbrain + Entorhinal + Superior frontal | 181 |
| 6 | Midbrain + Supramarginal + Entorhinal + Superior frontal + Middle temporal | 182 |
| 7 | Midbrain + Supramarginal + Entorhinal + Superior frontal + Insula | 183 |
| 8 | Midbrain + Entorhinal + Superior frontal + Isthmus cingulate | 188 |
| 9 | Midbrain + Entorhinal + Superior frontal + Middle temporal | 189 |
| 10 | Midbrain + Entorhinal + Superior frontal + Pons + Isthmus cingulate | 192 |

**Footnote:** The most efficient data-driven combinations of ROIs to detect 30% reduction in atrophy at 12 months with 10% attrition rate were derived from linear mixed-effects models.

**Abbreviations:** CBS, corticobasal syndrome; MRI, magnetic resonance imaging.
